# EPLIN-β is a novel substrate of ornithine decarboxylase antizyme 1 and mediates cellular migration

**DOI:** 10.1242/jcs.260427

**Published:** 2023-06-16

**Authors:** Dan Li, Suat Peng Neo, Jayantha Gunaratne, Kanaga Sabapathy

**Affiliations:** ^1^ Division of Cellular & Molecular Research, Humphrey Oei Institute of Cancer Research, National Cancer Centre Singapore, Singapore 168583, Singapore; ^2^ Institute of Molecular & Cellular Biology, Agency for Science, Technology and Research (A*STAR), Singapore 138673, Singapore; ^3^ Department of Anatomy, Yong Loo Lin School of Medicine, National University of Singapore, Singapore 117594, Singapore

**Keywords:** Antizyme, EPLIN, Polyamines, Ubiquitin-independent degradation, Cell migration

## Abstract

Polyamines promote cellular proliferation. Their levels are controlled by ornithine decarboxylase antizyme 1 (Az_1_, encoded by *OAZ1*), through the proteasome-mediated, ubiquitin-independent degradation of ornithine decarboxylase (ODC), the rate-limiting enzyme of polyamine biosynthesis. Az_1_-mediated degradation of other substrates such as cyclin D1 (*CCND1*), DNp73 (*TP73*) or Mps1 regulates cell growth and centrosome amplification, and the currently known six Az_1_ substrates are all linked with tumorigenesis. To understand whether Az_1_-mediated protein degradation might play a role in regulating other cellular processes associated with tumorigenesis, we employed quantitative proteomics to identify novel Az_1_ substrates. Here, we describe the identification of LIM domain and actin-binding protein 1 (LIMA1), also known as epithelial protein lost in neoplasm (EPLIN), as a new Az_1_ target. Interestingly, between the two EPLIN isoforms (α and β), only EPLIN-β is a substrate of Az_1_. The interaction between EPLIN-β and Az_1_ appears to be indirect, and EPLIN-β is degraded by Az_1_ in a ubiquitination-independent manner. Az_1_ absence leads to elevated EPLIN-β levels, causing enhanced cellular migration. Consistently, higher *LIMA1* levels correlate with poorer overall survival of colorectal cancer patients. Overall, this study identifies EPLIN-β as a novel Az_1_ substrate regulating cellular migration.

## Teaser

**Summary:** EPLIN-β is degraded by ornithine decarboxylase antizyme 1 (Az_1_) in a proteasome-mediated, ubiquitination-independent manner. Az1 absence leads to elevated EPLIN-β levels, causing enhanced cellular migration.

## INTRODUCTION

Polyamines, which include putrescine, spermidine and spermine, are bound to and modulate the functions of negatively charged molecules, such as DNA and RNA, as well as negatively charged proteins (Igarashi and Kashiwagi, 2000). They are not only essential for normal cellular growth and differentiation, but also play an important role in cellular proliferation and in the development of cancers (Nowotarski et al., 2013). Elevated levels of polyamines are associated with multiple cancer types, including breast, colon, lung, prostate and skin cancers (Nowotarski et al., 2013), and, hence, polyamine levels need to be carefully regulated.

Ornithine decarboxylase (ODC, encoded by *ODC1*) is the rate-limiting enzyme regulating polyamine synthesis (Palanimurugan et al., 2004). This enzyme catalyzes the decarboxylation of ornithine to form putrescine, a common step involved in polyamine biosynthesis, leading to the generation of spermidine and spermine. High ODC activity is associated with rapid proliferation of normal and cancerous cells and tissues (Porter et al., 1987; Gerner and Meyskens, 2004). ODC is negatively regulated by the ornithine decarboxylase antizyme (referred to as Oaz or Az) family, which is encoded by three genes: *OAZ1* (Az_1_), *OAZ2* (Az_2_) and *OAZ3* (Az_3_) in humans (Ivanov et al., 1998; Chen et al., 2002; Snapir et al., 2009). The most predominant Az protein is Az_1_, which is ubiquitously expressed along with Az_2_, although at higher levels than the latter (Ivanov et al., 1998). Az_3_ is, however, only expressed in the testis (Ivanov et al., 2000).

Az proteins are expressed in a unique manner, as they are derived from two overlapping open reading frames (ORFs): ORF1 contains the translational start codon and also has a stop codon at a frameshift site, and ORF2 encodes most of the protein and lacks an initiation codon (Matsufuji et al., 1995). A +1 ribosomal frameshift results in the skipping of one nucleotide at the stop codon of ORF1 and leads to continued translation to the end of ORF2, which results in the production of functional full-length Az proteins (Matsufuji et al., 1995). Increased intracellular polyamine levels stimulate the +1 frameshift, which then negatively regulates ODC expression as a feedback mechanism to regulate polyamine levels in cells (Matsufuji et al., 1995; Rom and Kahana, 1994; Agostinelli et al., 2010; Coffino, 2001).

The enzymatically active ODC functions as a homodimer, but ODC monomers bind to Az proteins with higher affinity to form ODC–Az heterodimers (Matsufuji et al., 1995). Az binding promotes the degradation of ODC through the 26S proteasome, independent of ubiquitination (Murakami et al., 1992). Although all Az proteins are able to inhibit ODC activity and polyamine uptake, only Az_1_ induces ODC degradation (Murakami et al., 1992; Li and Coffino, 1993; Mangold and Leberer, 2005). The role of Az_2_ in ODC degradation is controversial (Chen et al., 2002; Murakami et al., 1992; Zhu et al., 1999), and Az_3_ is unable to induce ODC degradation (Snapir et al., 2009). Therefore, Az_1_-mediated downregulation of ODC and the consequential inhibition of polyamine biosynthesis are crucial processes in polyamine homeostasis.

Az_1_ activity is regulated by an inhibitory interaction with Az inhibitor (AZIN, encoded by *AZIN1*), which is highly similar to ODC (Qiu et al., 2017; Nilsson et al., 2000). Given the similarity to ODC, AZIN competitively binds to Az, thereby relieving ODC from Az-mediated degradation. Thus, the Az/AZIN ratio determines cellular polyamine homeostasis, thereby regulating cellular growth (Olsen and Zetter, 2011). The levels of both Az_1_ and AZIN have been evaluated in several cancers and corresponding normal tissues, which indicate that Az_1_ levels are indeed reduced with a concomitant increase in AZIN levels in cancers (Olsen and Zetter, 2011), supporting a model in which a decrease in Az_1_ levels and/or activity promotes cancer development.

Besides ODC, five other proteins that are upregulated in cancers have been identified as Az_1_ substrates to date, including DNp73 (encoded by *TP73*), Aurora A (*AURKA*), cyclin D1 (*CCND1*), Mps1 and Smad1 (Lim and Gopalan, 2007; Newman et al., 2004; Kasbek et al., 2010; Gruendler et al., 2001; Dulloo et al., 2010). Our laboratory has previously shown that Az_1_ is a negative regulator of DNp73 (Dulloo et al., 2010), an oncoprotein of the p53 (*TP53*) tumor-suppressor family, that is overexpressed in a variety of cancers and the expression of which leads to resistance to a variety of chemotherapeutic drugs and metastasis (Müller et al., 2005; Steder et al., 2013). Az_1_-mediated DNp73 degradation also occurs in a ubiquitin-independent but proteasome-dependent manner and facilitates chemosensitivity (Dulloo et al., 2010), collectively suggesting that Az_1_ could regulate the expression of a larger set of substrates to prevent tumorigenesis.

In order to uncover the full repertoire of Az_1_ substrates, we undertook a proteomics approach using cells lacking Az_1_ expression and identified EPLIN-β as a novel Az_1_ substrate. EPLIN is an actin-binding protein encoded by the *LIMA1* gene. It is expressed as two isoforms: EPLIN-α, a 600 amino-acid protein, and EPLIN-β, a 760 amino-acid protein, generated from an alternative pre-mRNA splicing event (Maul and Chang, 1999). Both isoforms participate in regulating actin cytoskeleton and dynamics. They contain a LIM domain, which is a cysteine-rich domain composed of two zinc fingers and functions as a modular interface to facilitate protein–protein interactions (Kang et al., 2000; Sánchez-García and Rabbits, 1994; Schmeichel and Beckerle, 1994). Most LIM domain-containing proteins are frequently present in molecules responsible for cytoskeletal organization (Brown et al., 1996; Collins et al., 2015), and EPLINs are also involved in maintaining epithelial cell junctions (Maul and Chang, 1999; Maul et al., 2003; Abe and Takeichi, 2008; Song et al., 2002).

Loss of EPLIN-α expression has been implicated in the progression of various cancers, such as oral, breast and prostate cancers, in which its expression was either downregulated or completely abolished compared to normal tissues, indicating a tumor-suppressor role for EPLIN-α (Liu et al., 2012, 2016). On the contrary, increased levels of EPLIN-β have been noted in various cancers (Maul and Chang, 1999), suggestive of an oncogenic or growth-promoting role for EPLIN-β. Our results illustrate that although both isoforms are able to interact with Az_1_, only EPLIN-β is degraded by Az_1_ in a ubiquitin-independent manner. Functional analysis indicated that EPLIN-β is a positive regulator of cellular migration, being upregulated in and causal to the enhanced cellular growth and migration of Az_1_-null (Az_1_^−/−^) cells. These data therefore demonstrate that EPLIN-β is a novel Az_1_ substrate mediating cellular migration.

## RESULTS

### Generation of Az_1_-deficient cells

**Fig. 1.**
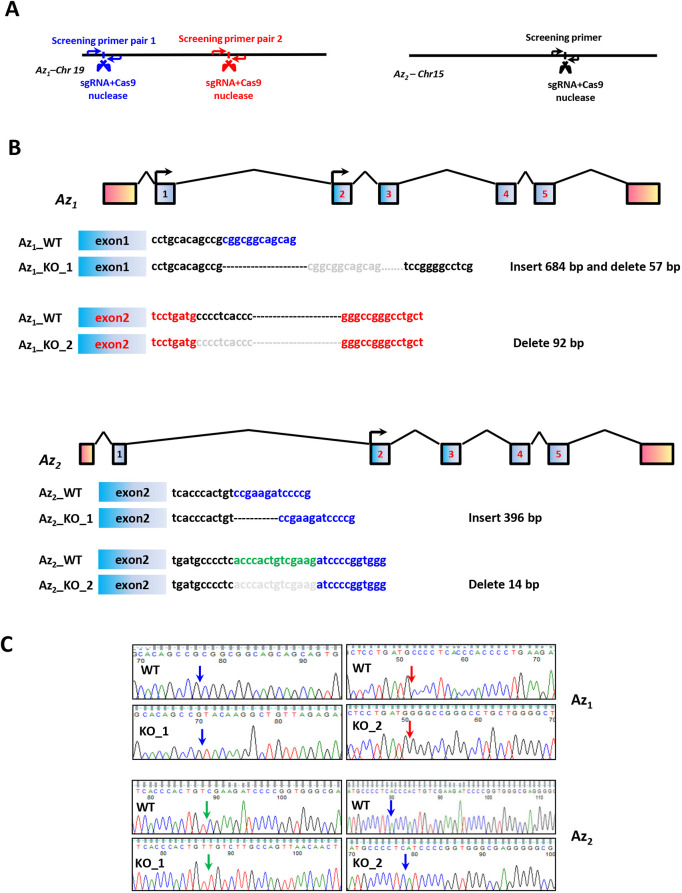
**Generation of Az_1_ and Az_2_ knockout cells.** (A) Schematic indicating the positions of the sgRNAs used for targeting. (B) The schematic shows deletions and insertions in two Az_1_ and Az_2_ clones used in this study. Bp, base pair. (C) Sequencing data for WT, Az_1_^−/−^ and Az_2_^−/−^ HCT116 cells are shown. Arrows indicate the insertion or deletion sites on the locus.

In order to identify novel Az_1_ substrates, we generated HCT116 human colorectal cells lacking Az_1_ using CRISPR-Cas9 genome editing. The aim was to identify proteins with elevated expression levels in the absence of Az_1_. Colorectal cells were chosen for the screen as polyamines are associated with the physiology of the human gut, and their levels are elevated in gastrointestinal tumors (Tofalo et al., 2019). Two pairs of guide (g) RNAs (lentiviral CRISPR constructs) targeting different coding regions of *OAZ1* were used. A schematic diagram of the location of the gRNA targeting site and the screening primer locations are shown in Fig. 1A. The first pair of gRNAs targeted ORF1 of *OAZ1*, whereas the second pair targeted ORF2. gRNAs targeting ORF1 resulted in a 684-nucleotide insertion at the 238 nucleotide position within the CRISPR-Cas9 cutting region, and a consecutive 57-nucleotide deletion (corresponding to nucleotides 126–182) in one clone (Fig. 1B). The second pair of gRNAs led to the deletion of 92 nucleotides between nucleotides 1963 and 2054 (Fig. 1B,C, upper panel) in another clone. Sequencing analyses were performed to confirm these alterations in both clones (Fig. 1B,C, upper panel). *OAZ2* was targeted (Fig. 1A) and two representative clones with an insertion of 396 nucleotides and a deletion of 14 nucleotides were obtained (Fig. 1B,C, lower panel).

### Identification of EPLIN-β as an Az_1_ substrate

**Fig. 2.**
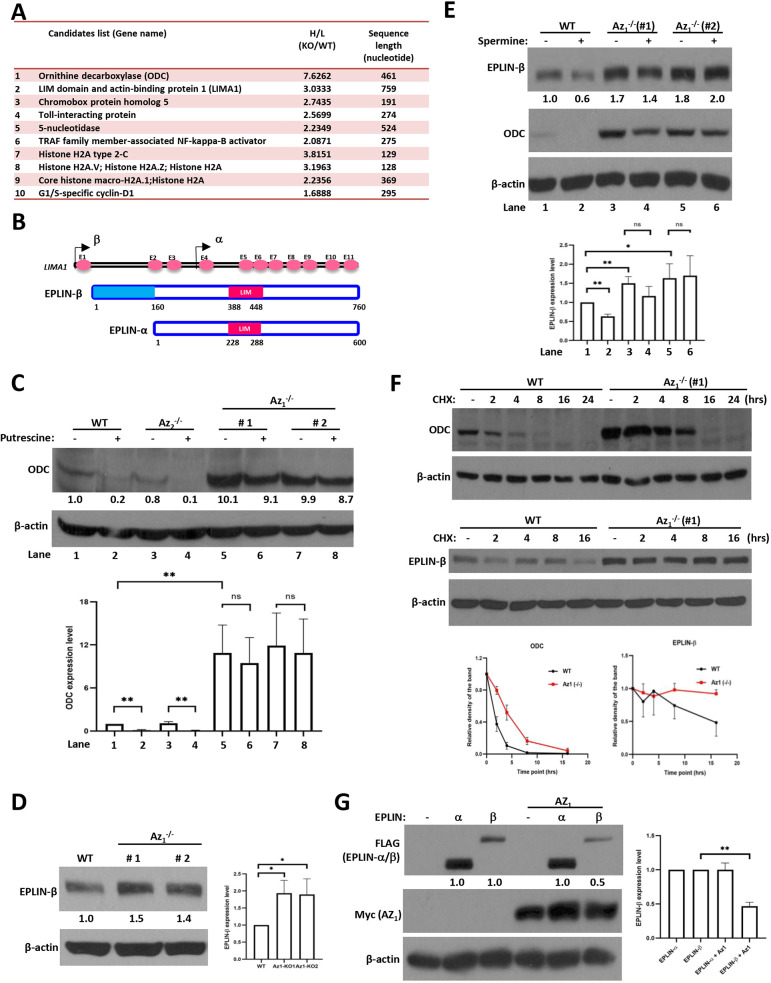
**EPLIN-β is a novel substrate of Az_1_.** (A) The list shows the top nine candidates, along with cyclin D1, identified from the SILAC analysis. (B) Schematic diagram of *LIMA1* gene and structure of the EPLIN-α and EPLIN-β isoforms. The *LIMA1* gene contains 11 exons and ten introns. EPLIN-β has an additional 160 amino acids at its amino-terminal, which are absent in EPLIN-α. (C,D) HCT116 WT, Az_1_-KO, and Az_2_-KO cells were treated with 2.5 µM putrescine for 24 h (C) or were used without any treatment (D). The cells were then harvested and lysed, and immunoblotting was performed to determine the expression of the various proteins indicated. Expression levels of ODC (C) and EPLIN-β (D) were quantified relative to β-actin and are indicated below the blots and in the histograms. The expression levels of the proteins in untreated WT cells were used as a reference (e.g. 1.0). (E,F) WT and Az_1_-KO cells were treated with 50 µM spermine for 24 h (E), or with 100 µg/ml cycloheximide (CHX) for the indicated time periods (F), and were harvested and analyzed by immunoblotting. (G) H1299 cells were transfected with EPLIN-α- and EPLIN-β-expressing plasmids without or with the Az_1_-expressing plasmid. At 24 h post transfection, the cells were harvested and analyzed by immunoblotting. Multiple blots were run with the same lysates (and same amounts) for detection with the various antibodies (e.g. in E,G), and a representative anti-actin blot for loading control is shown. All experiments were repeated three times independently and representative blots are shown. Graphs show the mean±s.d. and statistical analyses of quantifications, based on three independent experiments. Statistical comparisons between two groups were carried out by two-tailed unpaired Student's *t*-test. ns, not significant; *P<0.05; **P <0.01.

Based on the hypothesis that increased levels of Az_1_ substrate proteins could be identified from cells lacking Az_1_, we employed the quantitative stable isotope labeling using amino acids in cell culture (SILAC) proteomics methodology (Ong et al., 2002) and compared the differential expression of proteins between Az_1_^−/−^ knockout (KO) cells and their wild-type (WT) controls. Changes in expression of greater than twofold between heavy and light proteins (H/L ratios) were considered significant (Duan et al., 2010). The initial analysis led to the identification of several proteins that were significantly differentially expressed between Az_1_^−/−^ cells and their WT controls, among which ODC ranked first, validating our experimental approach (Fig. 2A). The ratios of the expression of these proteins between KO and WT cells (KO/WT ratios) are shown in Table S1. In addition, another well-known Az_1_ substrate, cyclin D1, was also identified in the analysis, albeit below the H/L ratio of 2, indicating that many more bona fide substrates could be identified at lower thresholds. Of note, EPLIN (*LIMA1*) was second on the list. Hence, we focused our efforts in characterizing the role of Az_1_ in the regulation of EPLIN. As aforementioned, EPLIN is expressed as two isoforms generated from an alternative pre-mRNA splicing event (Fig. 2B). Interestingly, our SILAC analysis identified that only EPLIN-β, but not EPLIN-α, was upregulated in the absence of Az_1_, indicating that the additional sequences in EPLIN-β might be critical for Az_1_-mediated regulation.

To validate whether EPLIN-β is indeed regulated by Az_1_, we undertook several approaches. Firstly, we quantified the expression of ODC as well as EPLIN-β levels in Az_1_^−/−^ cells. Baseline ODC levels were low in HCT116 WT cells, but were significantly elevated in two Az_1_^−/−^ clones (Fig. 2C), further establishing the utility of the Az_1_^−/−^ cells in the study of its substrates. However, the absence of Az_2_ did not affect ODC levels, highlighting a dominant role for Az_1_ in ODC regulation, as reported previously (Murakami et al., 1992). Furthermore, treatment with polyamines (e.g. putrescine), which induces the frameshift of endogenous Az, caused a further decrease in ODC levels only in WT and Az_2_^−/−^ cells, but had no major effects in Az_1_^−/−^ cells (Fig. 2C), demonstrating the importance of Az_1_ in ODC regulation.

The expression of EPLIN-β was also upregulated in the absence of Az_1_ (Fig. 2D), but not in the absence of Az_2_ (Fig. S1A), without any significant impact on *EPLIN-β* mRNA levels (Fig. S1B). In addition, EPLIN-β expression was also examined by immunofluorescence, and its expression was noted to be significantly increased in Az_1_^−/−^ cells compared to that in WT cells (Fig. S2, top panel; experimental replicates for Fig. S2 are provided in Fig. S13), supporting the immunoblotting data. In addition, although we were unable to detect an increase in ODC fluorescence intensity in Az_1_^−/−^ cells, there was a change in its localization, being found both in the cytoplasm and nucleus in Az_1_^−/−^ cells, unlike in WT cells in which it was predominantly nuclear (Fig. S2, middle panel). Thus, by both assays, the absence of Az_1_ led to an increase in EPLIN-β expression.

Moreover, although treatment of WT cells with polyamines (e.g. spermine) led to a decrease in EPLIN-β levels, this was not the case in cells lacking Az_1_, in which EPLIN-β levels remained relatively unchanged (Fig. 2E), further confirming that EPLIN-β levels are indeed regulated in an Az_1_-dependent manner. In addition, treatment of cells with cycloheximide (CHX) to block protein synthesis and subsequent chase indicated that the decay of EPLIN-β and ODC proteins was significantly delayed in the absence of Az_1_ (Fig. 2F), suggesting that the effects of Az_1_ on EPLIN-β and ODC levels were at the post-transcriptional level, leading to their extended half-lives.

Finally, we expressed cDNAs encoding either EPLIN-α or EPLIN-β alone or with Az_1_ in H1299 cells to evaluate the direct impact of Az_1_ overexpression on EPLIN levels. Co-expression with Az_1_ led to a decrease in EPLIN-β but not EPLIN-α levels (Fig. 2G). However, co-expression with Az_2_ did not affect EPLIN-β expression (Fig. S1C). These data collectively establish that EPLIN-β, but not EPLIN-α, is a target of Az_1_-mediated degradation.

### The LIM domain is required for EPLIN-β–Az_1_ interaction and EPLIN-β is degraded in a ubiquitin-independent manner

**Fig. 3.**
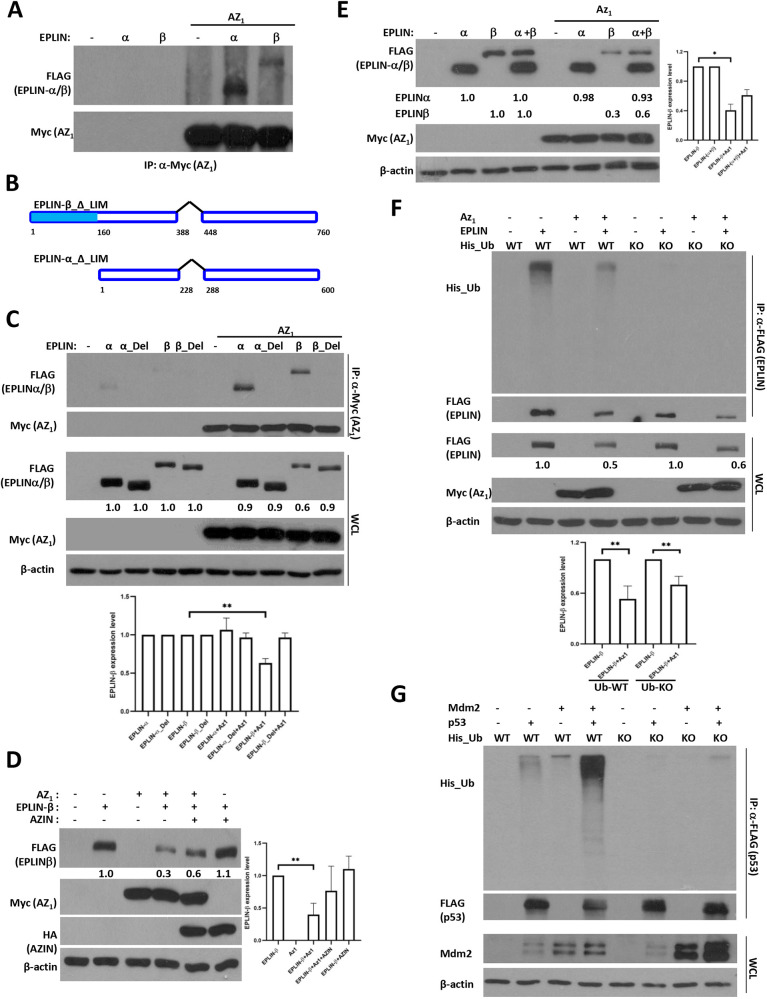
**Az_1_ interacts with EPLINs through their LIM domain.** (A–E) H1299 cells were transfected with the indicated plasmids, and cellular lysates collected 24 h post transfection were used either for immunoprecipitation (IP) with an anti-Myc antibody (A,C), or for direct immunoblotting analysis (D,E). A schematic structure of the EPLIN-α and EPLIN-β plasmids used with LIM domain deletion (‘Del’) is shown in B. WCL, whole-cell lysates. (F,G) H1299 cells were transfected with the indicated plasmids and used for IP analysis as described above. Multiple blots were run with the same lysates (and same amounts) for detection with the various antibodies (e.g. in C–F), and a representative anti-actin blot for loading control is shown. All experiments were repeated three times (except that shown in E, which was repeated twice) independently and representative blots are shown. Graphs show the mean±s.d. and statistical analyses of quantifications of all the independent experiments. Statistical comparisons between two groups were carried out by two-tailed unpaired Student's *t*-test. *P<0.05; **P<0.01.

As all the reported substrates of Az_1_ interact with it, we next evaluated whether EPLIN also interacts with Az_1_. As shown in Fig. 3A, both EPLIN-α and EPLIN-β interacted with Az_1_ when co-expressed. As both EPLIN-α and EPLIN-β contain a centrally located LIM domain, which might mediate self-dimerization or allow EPLIN to interact with other proteins (Fig. 3B) (Maul et al., 2003; Schmeichel and Beckerle, 1994), we hypothesized that EPLIN might interact with Az_1_ through its LIM domain. To evaluate this possibility, we generated cDNAs encoding EPLIN-α and EPLIN-β with LIM domain deletions, which were co-expressed with Az_1_. The absence of the LIM domain abrogated binding of both EPLIN isoforms to Az_1_ (Fig. 3C), suggesting that the LIM domain of EPLIN is critical for its interaction with Az_1_. Nonetheless, EPLIN-β lacking the LIM domain was refractory to Az_1_-mediated degradation (Fig. 3C), emphasizing the importance of the Az_1_–LIM interaction for the degradation of EPLIN-β. Moreover, co-expression with AZIN led to a partial rescue of EPLIN-β degradation by Az_1_ (Fig. 3D), similar to observations with DNp73 and Cyclin D1 (Newman et al., 2004; Dulloo et al., 2010). Additionally, co-expression of EPLIN-α along with EPLIN-β had a similar effect as AZIN, reducing EPLIN-β degradation by Az_1_ (Fig. 3E), suggesting a competitive model between the two isoforms resulting in the protection of EPLIN-β in the presence of EPLIN-α.

Using purified proteins, we explored whether the interaction between these two partners was direct. Based on our analyses, we were not able to observe a direct interaction between these two proteins, or with the LIM domain alone (data not shown). Thus, we believe that the interaction between Az_1_ and EPLIN- β is likely indirect, in a ternary complex or, alternatively, in a way that the purified proteins do not reflect the conformation *in vivo* to reveal the binding.

Lastly, we evaluated whether Az_1_-mediated EPLIN-β degradation occurs independent of ubiquitination, similar to other Az_1_ substrates. To that end, we co-expressed Az_1_ and EPLIN-β along with either WT ubiquitin, or one in which the seven lysine residues required for ubiquitin chain formation were substituted to arginine residues (i.e. Ubi-KO) (Stringer and Piper, 2011). EPLIN-β levels were reduced in the presence Az_1_ irrespective of the ubiquitin status (Fig. 3F). As a positive control, we used Mdm2-mediated degradation of p53 to demonstrate the dependence on ubiquitin chain formation (Fig. 3G), as reported previously (Oliner et al., 1993; Haupt et al., 1997). These data together demonstrate that EPLIN-β is degraded by Az_1_ in a ubiquitin-independent manner.

### Silencing EPLIN-β expression rescues accelerated cellular growth and migration of Az_1_^−/−^ cells

**Fig. 4.**
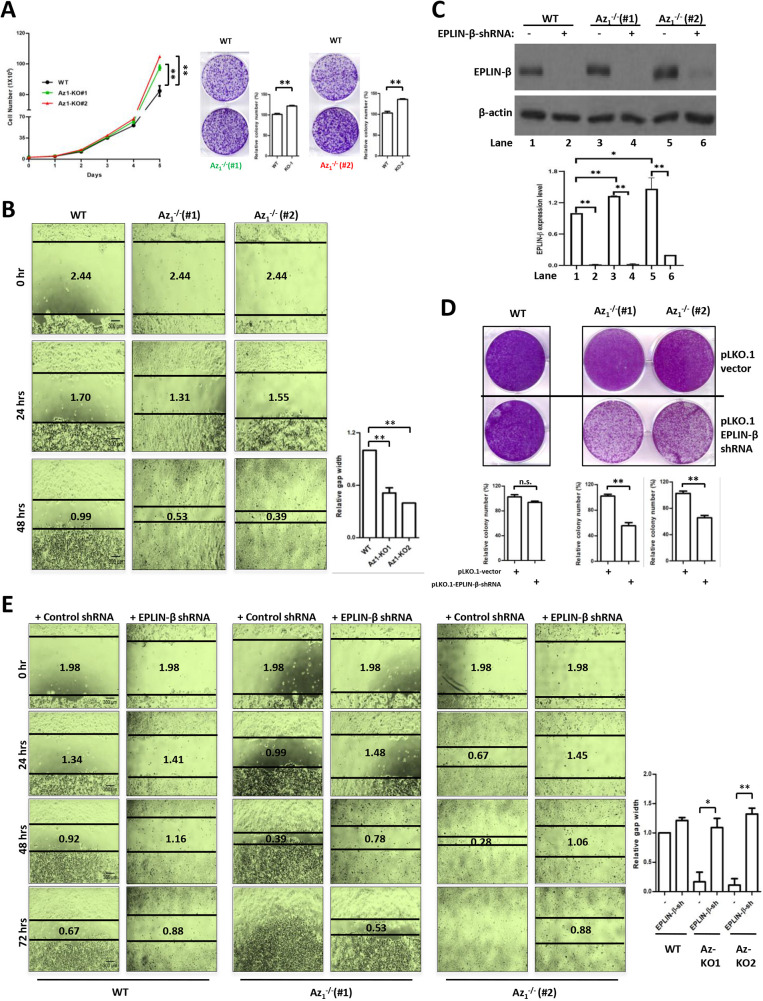
**Absence of Az_1_ promotes cell proliferation and migration.** (A) Growth rates of two Az_1_-KO clones were compared with those of WT HCT116 cells over several days (left). The *x*-axis represents time (days) after cell seeding. The cells were seeded and grown for 2 weeks prior to Crystal Violet (0.5% w/v) staining to determine colony growth (right). (B) Monolayers of WT or Az_1_-KO cells were scraped with a 200-µl sterile pipette tip to generate open wounds, and images were captured at 0, 24 and 48 h (4× magnification) to visualize the wound area that is covered. The distances between the edges were determined and are indicated in mm. The average values of the wound closure from three images at the 48 h time point are shown beside the images. Scale bars: 300 μm. (C–E) EPLIN-β expression was silenced using the control pLKO.1 vector (−) and pLKO.1 EPLIN-β shRNA (+) in WT and Az_1_-KO cells and the expression levels are shown in C. The cells were used in cellular colony formation (D) and in wound healing assays (E). The average values of the wound closure from three images at the 72 h time point are shown beside the images. Scale bars: 300 μm. All experiments were repeated three times independently and representative blots/images are shown. Experimental replicates of the images shown in B,E are given in Fig. S12. Graphs show the mean±s.d. and statistical analyses of quantifications, based on three independent experiments. Statistical comparisons between two groups were carried out by two-tailed unpaired Student's *t*-test. n.s., not significant; *P<0.05; **P<0.01.

To determine whether the Az_1_–EPLIN-β interaction has a functional role in cellular growth regulation, we first evaluated the effects of Az_1_ deficiency on cellular growth and migration. Compared to control cell numbers, Az_1_^−/−^ cell numbers increased significantly, leading to increased cellular colony formation (Fig. 4A). Moreover, the absence of Az_1_ led to accelerated cellular migration in wound-healing assays (area of open wound after 48 h: control cells, 0.99 mm; Az_1_^−/−^ clone 1, 0.53 mm; Az_1_^−/−^ clone 2, 0.39 mm) (Fig. 4B), together confirming the inhibitory effects of Az_1_ on cellular growth and motility.

We next examined whether this observed growth advantage could be due to elevated EPLIN-β levels. Although the majority of the previous studies indicated a putative tumor suppressor role of EPLINs, the roles of each individual isoform have not been fully elucidated. Hence, we silenced the expression of EPLIN-β in parental and Az_1_^−/−^ clones (Fig. 4C). Silencing EPLIN-β led to a significant reduction in the growth of colonies in both Az_1_^−/−^ clones, whereas the effect was marginal in HCT116 WT cells (Fig. 4D), indicating that elevated EPLIN-β expression in the absence of Az_1_ indeed contributed to its accelerated growth. Moreover, a similar reversal of the accelerated migration of Az_1_^−/−^ cells was noted in the wound healing assays (area of open wound after 48 h: control cells with control shRNA, 0.92 mm; Az_1_^−/−^ clone 1 with control shRNA, 0.39 mm; Az_1_^−/−^ clone 2 with control shRNA, 0.28 mm; control cells with EPLIN-β shRNA, 1.16 mm; Az_1_^−/−^ clone 1 with EPLIN-β shRNA, 0.78 mm; Az_1_^−/−^ clone 2 with EPLIN-β shRNA, 1.06 mm) (Fig. 4E). These results further illustrate that the increased growth and migration observed in Az_1_^−/−^ cells is due to the upregulation of EPLIN-β.

### High levels of EPLIN-β prognosticate poorer survival rates

**Fig. 5.**
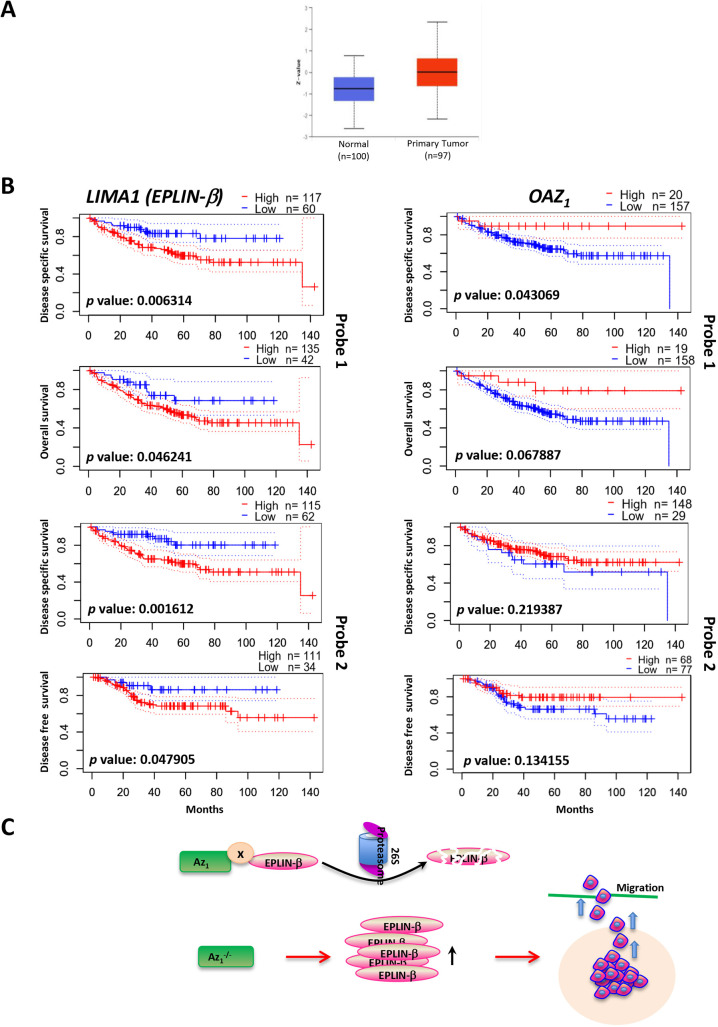
***LIMA1* or *OAZ1* expression and prognosis among colorectal cancer patients.** (A) Higher *LIMA1* gene expression was noted in primary tumor tissues compared to that in normal tissues from colorectal cancer (CRC) patients, based on the analysis of the UALCAN database (http://ualcan.path.uab.edu/index.html). Boxes indicate the 25–75th percentile, whiskers show the highest and lowest values, and the median is marked with a line. (B) Correlation between *LIMA1* (in this case, representing EPLIN-β only) (left) and *OAZ1* (right) expression with CRC patients' prognosis was determined from dataset GSE17536 using PrognoScan. Kaplan–Meier plots are shown for overall survival, disease specific survival, and disease free survival for two specific probes of each gene. The same dataset was used in both cases. Patients were segregated based on high (red) or low (blue) expression levels. The number of patients in each group is shown at the top of each graph. The log-rank test was applied in the Kaplan–Meier survival examination. (C) Working model. EPLIN-β is a novel substrate of Az_1_. The interaction of EPLIN-β with Az_1_ (probably through other proteins, indicated as ‘x’) results in its degradation in a ubiquitination-independent and proteasome-dependent manner. Absence of Az_1_ leads to increased EPLIN-β levels, which promote the migration of HCT116 cells.

The above data suggest that EPLIN-β might function as a cellular growth promoter rather than a tumor suppressor. To assess its expression in cancers, we evaluated and found that *LIMA1* expression was higher in tumor tissues compared to that in normal tissues in colorectal cancers, based on the University of Alabama at Birmingham Cancer data analysis Portal (UALCAN) website (http://ualcan.path.uab.edu/index.html) (Fig. 5A). Furthermore, Kaplan–Meier analysis of survivability in a colorectal carcinoma dataset (GSE17536) from PrognoScan (Mizuno et al., 2009) indicated that higher levels of *LIMA1* (EPLIN-β only) are correlated with a significantly lower survival probability (Fig. 5B, left panel). Interestingly, higher *OAZ1* levels correlated with a higher survival probability in the same dataset (Fig. 5B, right panel), albeit with much lower significance. These data suggest that EPLIN-β, which enhances cancer cell growth and migration, is indeed associated with poorer patient survival.

## DISCUSSION

This report describes EPLIN-β as a novel Az_1_ substrate. This work expands the repertoire of Az_1_ substrates to seven and also suggests the existence of many more substrates that are yet to be identified and characterized. Our proteomics analysis identified around ten substrates in HCT116 colorectal cells that were upregulated significantly (H/L>2) in the absence of Az_1_, of which we characterized EPLIN-β here, and the others are being currently investigated. We envisage that there might be other substrates being regulated in a cell-type-specific and context-dependent manner, and these are being explored in our laboratory. The growing list of Az_1_ substrates prompts us to speculate that Az_1_-mediated protein degradation might be a yet underexplored regulatory mechanism that could be critical in the regulation of cellular growth, and thus in cancer development, as well as in other physiological and pathological processes.

Polyamines are ubiquitously produced, and their levels are relatively high in rapidly growing cells. In particular, increased polyamines levels have been shown to directly correlate with disease activity and tumor burden (Soda, 2011; Durie et al., 1977). Polyamines lead to the synthesis of full-length functional antizymes, which then negatively regulate polyamine production through the binding and proteasome-mediated degradation of ODC, the rate-limiting enzyme involved in polyamine biosynthesis (Kahana, 2018).

ODC, the classical bona fide substrate of Az_1_, has been used to study the mechanistic basis of Az_1_-mediated degradation. Binding of Az_1_ to ODC leads to disruption of ODC homodimers, leading to the exposure of the C-terminal tail, which targets ODC to the proteasome in a ubiquitin-independent manner (Palanimurugan et al., 2004; Pegg, 2006; Almrud et al., 2000; Li and Coffino, 1993). For a long time, no other substrates had been identified, but five other substrates (Mps1, Smad1, cyclin D1, Aurora A and DNp73) were then found to be degraded in an Az_1_-dependent manner. All of them have been shown to bind to Az_1_ and are targeted for degradation in a ubiquitin-independent manner (Lim and Gopalan, 2007; Dulloo et al., 2010; Newman et al., 2004; Kasbek et al., 2010; Gruendler et al., 2001).

Interestingly, a few features appear to be common among all the seven substrates identified thus far: they act as homodimers or can heterodimerize with highly identical isoform variants, and they are all involved in promoting cellular proliferation and/or migration, being overexpressed in a variety of cancers (D'Assoro et al., 2016; Alao, 2007; Daniel et al., 2011; Chen et al., 2021; Yokomizo et al., 1999). All seven substrates of Az_1_, including EPLIN-β, are negatively regulated by Az_1_, consistent with the growth inhibitory properties of the latter. Nevertheless, whether Az_1_-dependent degradation is indeed a relevant mechanism of proteolysis is unclear owing to the lack of further insights into its substrates. Our work was initiated to explore the Az_1_ substratome using Az_1_^−/−^ HCT116 colorectal cells. The absence of Az_1_ expression expectedly led to elevated levels of ODC, which was also identified as the top candidate in the SILAC analysis. This proteomics analysis also identified a list of proteins that are differentially expressed in the absence of Az_1_, suggesting that the Az_1_ substratome might be much larger than expected, and is the subject of our future investigations.

Similar to the other Az_1_ substrates, EPLIN-β was able to interact with Az_1_ and was degraded in an Az_1_-dependent manner without the need for ubiquitination. Co-expression with Az_1_ or treatment with polyamines was sufficient for EPLIN-β degradation in an Az_1_-dependent manner. Furthermore, the enhanced cellular growth and migration phenotypes observed in the absence of Az_1_ and that were reversed by silencing of EPLIN-β demonstrate the functional interaction between Az_1_ and EPLIN-β. Consistently, we also found that high EPLIN-β levels or lower Az_1_ levels prognosticated for poorer survival, further confirming the functionality of the data in the clinical context.

EPLIN-β is an actin-binding protein, and its negative regulation by Az_1_ extends a role for the latter in cellular migration, expanding the repertoire of tumor suppressive functions of Az_1_. Az_1_ has been suggested to be a tumor suppressor, with its expression being reduced in many cancer types (Abe and Takeichi, 2008). Previous analysis of cyclin D1 as an Az_1_ substrate has suggested its role in cellular proliferation and growth (Newman et al., 2004). Consistently, Az_1_^−/−^ cells showed enhanced cell growth, and we also noticed that they migrated faster in cellular wound healing assays compared to their WT counterparts. Furthermore, silencing of EPLIN-β reversed the enhanced migration of Az_1_^−/−^ cells, further demonstrating an important role of Az_1_ in regulating cellular motility.

Among the two EPLIN isoforms, EPLIN-α expression is significantly downregulated in several cancers (compared to normal tissues), leading to enhanced migration or invasion capabilities (Maul and Chang, 1999). Overexpression of EPLIN-α leads to inhibition of cellular growth (Sanders et al., 2011; Jiang et al., 2008). Hence, EPLINs were generally considered as tumor suppressors. However, it is to be noted that cancers also exhibit a concomitant increase in EPLIN-β expression (Maul and Chang, 1999), although it has not been clarified whether the reduction in EPLIN-α or the increase in EPLIN-β, or a combination of both, is causal to the enhanced cellular migration. Our results indicate that, at least in HCT116 colorectal cancer cells, EPLIN-β plays a pro-growth role, as opposed to the established tumor-suppressor functions of EPLIN-α (Sanders et al., 2011; Jiang et al., 2008). Consistently, analysis of several human colorectal cancer gene expression datasets showed that survival rates were significantly reduced among the patients with tumors expressing relatively higher levels of *LIMA1* (denoting EPLIN-β) compared to those with lower *LIMA1* levels. This further indicates that EPLIN-α expression is either downregulated or undetectable in colorectal cancers, whereas EPLIN-β expression is upregulated, consistent with previous observations (Maul and Chang, 1999).

Several points are worth highlighting based on our results. Firstly, as with other substrates, binding of Az_1_ alone is insufficient, although it is necessary for EPLIN-β degradation. As such, the other EPLIN-α isoform, which is also able to bind to Az_1_, is not degraded by Az_1_. We speculate that although the binding region is common to both isoforms, the additional 160 amino acids of EPLIN-β are critical for degradation. Given the lack of these 160 amino acids in EPLIN-α, it is not degraded, albeit being able to bind. Therefore, structural predictions of the N-terminus of EPLIN-β were performed using the ‘AlphaFold Protein Structure Database’ (Jumper et al., 2021). It was found that the extra 160 amino acids of EPLIN-β contain an intrinsically disordered region (data not shown). As proteins with terminal or internal intrinsically disordered segments are easily recognized by the 26S proteasome for degradation (van der Lee et al., 2014), we assume that this disordered region in the N-terminus of EPLIN-β, which is absent in EPLIN-α, contributes to the degradation of EPLIN-β by 26S proteasome.

This observation is similar to the p73 proteins, of which DNp73, but not the TAp73 isoform, is degraded by Az_1_, albeit both being able to bind Az_1_ (Dulloo et al., 2010). This indicates that the presence of additional domains is required for the degradation process. Although the mechanistic basis of Az_1_-mediated selective degradation of related proteins is unclear, we postulate that the additional regions are structurally altered upon Az_1_ binding, and thus lead to proteasomal degradation. This is the case for ODC, where the structure of ODC changes upon binding to Az_1_, resulting in the exposure of its C-terminus to the 26S proteasome (Wu et al., 2015). Interestingly, EPLIN-β does not contain any homologous regions to the Az_1_-binding interface of ODC (data not shown), suggesting that the mechanism of degradation of various Az_1_ substrates might be different.

Secondly, Az_1_, but not Az_2_, is the key mediator of EPLIN-β degradation. Both silencing and overexpression of Az_2_ did not alter EPLIN-β levels. Thus, although polyamines can induce the frameshifting of both Az_1_ and Az_2_, only the former appears to be the major regulator of protein abundance, at least in the cellular systems used in our study. Thirdly, it is to be noted that many of the newly identified substrates have a much lower H/L ratio compared to that of ODC. This probably provides an explanation as to why other Az_1_ substrates have not been identified thus far. Moreover, we detected the interaction between Az_1_ and EPLIN-β using overexpression systems, as it is technically difficult to detect interaction of endogenous proteins, as antibodies against processed full-length Az_1_ are currently not available commercially.

Finally, although EPLIN-β co-immunoprecipitated with Az_1_, we were not able to notice a binding using the respective purified proteins, as well as the LIM domain alone (data not shown). This suggests that although they do bind to each other, the interaction does not occur under isolated situations, indicating that either (1) the purified proteins are not correctly folded or in the correct conformation to bind each other, as we needed to use modified methods for purification of Az_1_/EPLIN-β, or (2) that some other unknown factors, particularly a protein co-factor, might be necessary for this interaction. Further biochemical studies are required to confirm whether this interaction is indeed direct or indirect.

Taken together, the data presented in this report evidently show that EPLIN-β is a novel substrate of Az_1_, mediating Az_1_-dependent cellular growth and migration (Fig. 5C). This study also highlights the existence of other substrates of Az_1_, warranting further exploration of the entire Az_1_ substratome, which could shed light on potential new targets that could be useful in clinical investigations. Moreover, this study also suggests that activation of Az_1_ could be a strategy to inhibit the growth and migration of tumor cells.

## MATERIALS AND METHODS

### Cell culture, reagents and transfection

p53-null human lung cancer H1299 cells, 293T cells and p53-proficient human colorectal carcinoma HCT116 cells were used in this study (obtained from American Type Culture Collection) (Dulloo et al., 2015). These three cell lines have been tested to be mycoplasma free. Cells were grown in Dulbecco's modified Eagle medium (DMEM; Hyclone) supplemented with 10% bovine fetal serum (FBS; Hyclone), 1% penicillin-streptomycin solution, 2 mM L-glutamine (Invitrogen, Carlsbad, CA, USA), 100 μM non-essential amino acids (Invitrogen) and 0.1 mM sodium pyruvate (Invitrogen), as described previously (Subramanian et al., 2015). The EPLIN-β knockdown cells were maintained in complete DMEM medium containing 0.5 µg/ml puromycin. Putrescine was 51799-100MG, lot BCBZ6097, Sigma-Aldrich; spermine was S3256-1G, lot BCBG8969V, Sigma-Aldrich.

The relevant plasmids were transfected using Lipofectamine 2000 (Invitrogen) in accordance with the manufacturer's instructions. Expression vectors (all in pcDNA3.0) expressing full length EPLIN-α, EPLIN-β, Az_1_, ODC or AZIN have been described previously (Li et al., 2010; Dulloo et al., 2010).

### SILAC

HCT116 parental cells were cultured with ‘heavy’ [SILAC Dulbecco’s modified Eagle’s medium (88364, Life Technologies), 10% dialyzed fetal bovine serum (26400044, Life Technologies), ^13^C_6_^15^N_2_ L-lysine-2HCl (143 mg/ml) (0.1%) (CNLM-291-0.5, Cambridge Isotopes), ^13^C_6_^15^N_4_ L-arginine-HCl (83 mg/ml) (0.1%) (CNLM-539-0.5, Cambridge Isotopes) and 1% penicillin-streptomycin solution (Hyclone, SV30010)] or ‘light’ [SILAC Dulbecco’s modified Eagle’s medium and 10% dialyzed fetal bovine serum, ^12^C_6_^14^N_2_ L-lysine-HCl (143 mg/ml) (0.1%) (L8662-25G, Sigma-Aldrich), ^12^C_6_^14^N_2_ L-arginine-HCl (83 mg/ml) (0.1%) (A8094-25G, Sigma-Aldrich) and 1% penicillin-streptomycin solution] medium and the corresponding Az_1_-KO cells were cultured with ‘light’ or ‘heavy’ medium for ∼4–5 doublings, at a cell density of around 20–30% confluence. The cells were harvested, and normalized protein extracts were used for SILAC analysis. LDS buffer (Invitrogen) and reducing agent (Invitrogen) were added to the lysates and boiled for 5 min. The proteins were then separated on 4–12% NuPage Novex Bis–Tris Gels (Invitrogen); stained with a colloidal blue staining kit (Invitrogen) and digested with trypsin using in-gel digestion procedures (Shevchenko et al., 2006).

### Mass spectrometry and data analysis

Tryptic peptides were analyzed using an EASY-nLC 1000 coupled to a Q Exactive Hybrid Quadrupole-Orbitrap (Thermo Fisher Scientific). The peptides were resolved and separated on a 50 cm analytical EASY-Spray column (ES803, Thermo Fisher Scientific) equipped with a pre-column over a 120 min gradient ranging from 8 to 38% of 0.1% formic acid in 95% acetonitrile/water at a flow rate of 200 nl/min. Survey full-scan mass spectrometry (MS) spectra (mass-to-charge ration or m/z, 310–2000) were acquired with a resolution of 70,000, an automatic gain control (AGC) target of 3×10^6^ and a maximum injection time of 10 ms. The top twenty most intense peptide ions in each survey scan were sequentially isolated to an AGC target value of 5×10^4^ with a resolution of 17,500 and fragmented using a normalized collision energy of 25. A dynamic exclusion of 10 s and isolation width of 2 m/z were applied. SILAC peptide and protein quantification was performed with MaxQuant version 1.5.0.30 (https://maxquant.org/) using default settings. Database searches of MS data were performed using UniProt human fasta (2017) (https://www.uniprot.org/proteomes/UP000005640) with tryptic specificity allowing a maximum of two missed cleavages, two labeled amino acids and an initial mass tolerance of 4.5 ppm for precursor ions and 0.5 Da for fragment ions. Cysteine carbamidomethylation was searched as a fixed modification, and N-acetylation and oxidized methionine were searched as variable modifications. Labeled arginine and lysine were specified as fixed modifications. Maximum false discovery rates were set to 0.01 for both proteins and peptides. Proteins were considered identified when supported by at least one unique peptide with a minimum length of seven amino acids. All mass spectrometry data have been deposited in the PRIDE repository with the accession number PXD039865.

### Immunoblot and immunoprecipitation analysis

Cell lysates were prepared in lysis buffer containing 0.5% Nonidet P-40 (NP-40) with protease inhibitor cocktail and dithiothreitol as described previously (Dulloo and Sabapathy, 2005). The total protein amounts were quantified and boiled in 4× SDS sample buffer, followed by separation on SDS polyacrylamide gels. Immunoblotting was performed with the following antibodies: anti-actin (A2066_.2 ml, lot 058M4812V, Sigma-Aldrich) and anti-ODC (O1136_.2 ml, lot 034M4836V, clone ODC-29) from Sigma-Aldrich (St. Louis, MO, USA); anti-FLAG (PA1-984B, lot WG319616) from Thermo Fisher Scientific; anti-EPLIN (sc-136399, lot H2117), anti-Myc (9E10) (sc-40, lot D0419) and anti-Mdm2 (sc-965, lot F2718; 1:200 for western blotting) from Santa Cruz Biotechnology, Dallas, TX, USA; anti-mouse secondary antibody (7076S, lot 36), anti-rabbit secondary antibody (7074S, lot 29), anti-HA-tag (C29F4) (3724S, lot 10) and anti-His-tag (27E8) (2366S, lot 14) from Cell Signaling Technology, Danvers, MA, USA. The antibody dilution factors are shown in Table S3. Uncropped images of the blots shown in Figs 2, 3 and 4 are shown in Figs S3, S6 and S9, respectively; experimental replicates of these blots are shown in Figs S4, S7 and S10, respectively, and uncropped images of the replicate blots are shown in Figs S5, S8 and S11, respectively. Uncropped images of the blots shown in Fig. S1 are shown in Fig. S9; experimental replicates of these blots in Fig. S10, and uncropped images of the replicate blots in Fig. S11.

Cell lysates, prepared as described above, were used for co-immunoprecipitation (IP) assays as described previously (Li et al., 2010). Briefly, cell lysates were immunoprecipitated with the indicated antibodies (both anti-FLAG and anti-Myc antibodies were used at a concentration of ∼5 μg/ml for IP) for 3 h at 4°C and then incubated with protein G-agarose (GE Healthcare Life Sciences) for 2 h at 4°C. Protein G-agarose was washed with NP-40 buffer three times and then analyzed by western blotting with the indicated antibodies.

### RNA-guided CRISPR-Cas9 nuclease-mediated Az knockout

To knockout Az_1_ or Az_2_ in HCT116 cells, CRISPR-Cas9 system was used as previously published (Sanjana et al., 2014). Briefly, sgRNAs targeting Az_1_ (Az_1_ gRNA1_Forward, 5′-caccgCTAAGCCTGCACAGCCGCGG-3′; Az_1_ gRNA1_Reverse, 5′-aaacCCGCGGCTGTGCAGGCTTAGc-3′; PCR screening primer_Forward1, 5′-GCAGCGGATCCTCAATAGCCAC-3′; PCR screening primer_Reverse1, 5′-CTTCTGGAAGCTTCGGACGG-3′; Az_1_ gRNA2_Forward, 5′-caccgGACTATTCCCTCGCCCACCT-3′; Az_1_ gRNA2_Reverse, 5′-aaacAGGTGGGCGAGGGAATAGTCc-3′; PCR screening primer_Forward2, 5′-GACCTGCATCATCTTCAGTTCC-3′; PCR screening primer_Reverse2, 5′-CTCAGGCAACGCCTGGGCTG-3′) or Az_2_ (Az_2_ gRNA_Forward, 5′-caccgCCACCGGGGATCTTCGACAG-3′; Az_2_ gRNA_Reverse, 5′-aaacCTGTCGAAGATCCCCGGTGGc-3′; PCR screening primer_Forward, 5′-CACCCTCTCTGTCTCTTGCAGTAG-3′; PCR screening primer_Reverse, CAGTGGAGTCTGAGAAAGCTCCAAC-3′) were designed using the online tool CRISPOR (http://crispor.tefor.net/) and cloned into the vectors lentiCRISPRv2 (Addgene plasmid #52961) using Bbs1 enzyme. After validation by DNA sequencing, the sgRNA vectors were co-transfected with the corresponding packaging plasmids into 293T cells, and the virus supernatant was prepared. HCT116 cells were then transduced with polybrene (4 mg/ml, Sigma-Aldrich) and viral supernatant, and selected on 0.5 μg/ml puromycin-containing medium for 10–12 days. Single colonies were randomly picked up into 96-well plates for genotyping by PCR and the knockouts were further validated by PCR using the Az_1_- or Az_2_-specific genomic primers.

### Protein half-life and ubiquitination assays

To determine EPLIN-β half-life, HCT parental and Az_1_ KO cells were seeded into six-well plates at ∼60% confluence. After 24 h, the cells were treated with the protein synthesis inhibitor cycloheximide (Sigma-Aldrich) for the indicated durations before harvesting and analysis by immunoblotting.

For EPLIN-β ubiquitination analysis, H1299 cells were transfected with His-ubiquitin (pCDNA3.0-6xHis-ubiquitin, cloned in our laboratory), Myc-Az_1_ (as above) or FLAG-EPLIN-β (as above). After 24 h of transfection, cells were lysed in NP-40 lysis buffer and then incubated with anti-FLAG antibody (Thermo Fisher Scientific; 5 μg/ml) for 3 h and protein G-agarose beads for 2 h at 4°C. After washing three times, the ubiquitinated EPLIN-β was detected by immunoblotting using anti-His monoclonal antibody.

### Immunofluorescence analysis

HCT116 parental and Az_1_-KO cells were seeded into six-well plates with glass coverslips. The next day, the permeabilized cells were blocked for 1 h with PBS supplemented with 2% bovine serum albumin and 5% FBS, and incubated with primary anti-ODC and anti-EPLIN antibodies (as above) antibodies at 4°C overnight, at 1:200 dilution (Phang et al., 2015). After washing, cells were incubated with secondary antibodies [goat anti-mouse IgG, IgM (H+L), secondary antibody, Alexa Fluro^TM^ 488, A11001, lot: 1397999, Thermo Fisher Scientific] for 1 h at room temperature. Images were visualized and captured using a Nikon Motorized Upright microscope 90i (NIS-Elements AR3.0, Japan).

### RNA extraction and real-time PCR

Total RNA was prepared from cells using TRIzol reagent (Invitrogen) according to the manufacturer's instructions. Then, 1.5–2 µg of total RNA was reverse transcribed into cDNA using SuperscriptII (Invitrogen). The sequences of real-time PCR primers and semi-quantitative reverse transcription-PCR analysis are the same as described (Subramanian et al., 2015).

### Cloning of shRNAs

The shRNA for EPLIN-β was cloned into pLKO.1 vector (Addgene 8453). Briefly, two restriction enzymes were used to linearize the vector followed by gel extraction. The insert shRNAs consist of two oligonucleotides that are complementary (forward and reverse primers listed in Table S2). When the two oligonucleotides annealed together, they contained the appropriate overhangs to allow cloning into the vector. The pLKO.1 vector was used as the control. The shRNAs were co-transfected with the corresponding packaging plasmids into 293T cells, and the virus supernatant was prepared. After 2 days of infection, the cells were under puromycin selection for 1 week before seeding for the experiments (Jin et al., 2018).

### Cellular colony formation and scratch-wound healing (migration) assays

HCT116 cells – WT, Az_1_ knockout or Az_1_ knockout cells, with or without EPLIN-β knock-down cells (1000 cells per well) – were plated on six-well plates and grown for 2 weeks. After rinsing three times with PBS, cells were fixed with 4% paraformaldehyde in PBS, and stained with 1% Crystal Violet.

Similarly, the above cells were seeded in 24-well plates at a density of 1.6×10^4^ cells/well in complete DMEM and cultured to confluence. The cells were then serum-starved overnight in DMEM, and the confluent cell monolayers were scraped with a yellow pipette tip to generate scratch wounds and were washed twice with Opti-MEM (Invitrogen) to remove cell debris (Zhao et al., 2011). Cells were incubated at 37°C with the medium containing 0.5 µg/ml puromycin. Time-lapse images were captured at 0, 24, 48 and 72 h time points in the same position using a Nikon Eclipse TE2000-5 microscope.

### Prognostic analysis of *LIMA1* and *OAZ1* genes

We checked the survival status of colorectal cancer patients by using PrognoScan (Mizuno et al., 2009) as it has features of a large collection of publicly available cancer microarray datasets with clinical annotation. The Kaplan–Meir plots of *LIMA1* and *OAZ1* genes were analyzed by using it.

### Statistical analysis

The differences in mean values were considered significant at *P*-values <0.01 (**) and <0.05 (*). The log-rank test was applied in the Kaplan–Meier survival examination. Statistical comparisons between two groups were carried out by two-tailed unpaired Student's *t*-test. A two-tailed *P*-value <0.05 was considered significant.

## Supplementary Material

10.1242/joces.260427_sup1 Supplementary information
