# Supplementary material for: EPLIN-β is a novel substrate of ornithine decarboxylase antizyme 1 and mediates cellular migration
Source: J Cell Sci. 2023 Jun 16;136(12):jcs260427. doi: 10.1242/jcs.260427 (PMC10281260; doi:10.1242/jcs.260427)
Supplement: Supplementary information [file joces-136-260427-s1.pdf]

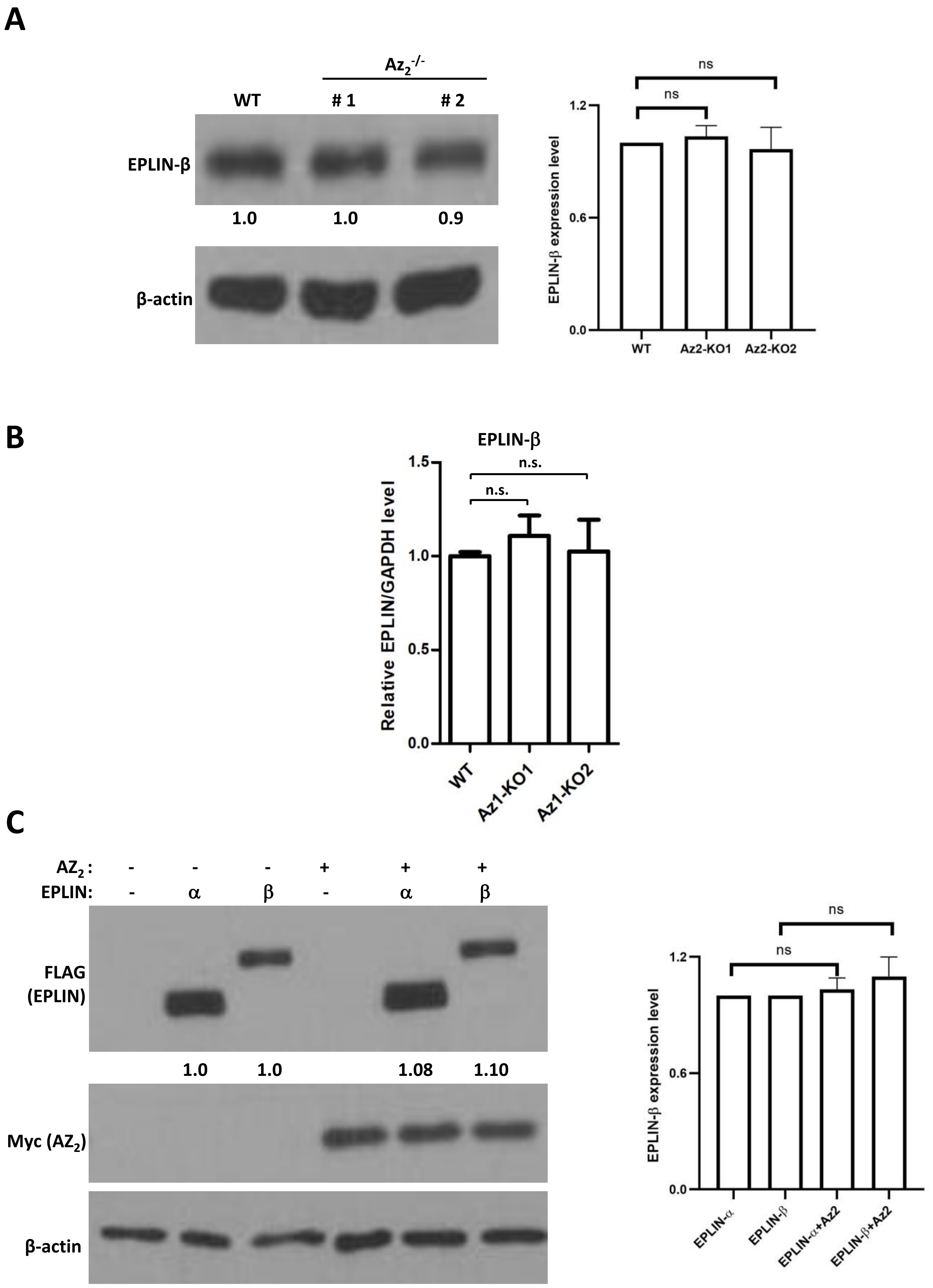

Fig. S1. Analysis of the effects of Az_1 and Az_2 on EPLIN-β

(A-B) HCT116 parental (WT) and Az_2-KO cells (A) or Az_1-KO cell clones (B) were harvested and used for immunoblotting analysis (A), or the RNA was used to determine *EPLIN-β* by real-time qPCR analysis (B). (C) H1299 cells were transfected with the indicated plasmids, and harvested 24 hrs post-transfection and used for immunoblotting analysis. n.s.: not significant.

All experiments were repeated three times (except Fig. S1B, twice) independently and representative blots/images are shown. Graphs show statistical analyses of quantifications, based on all independent experiments. Statistical comparisons between two groups were carried out by Student's *t*-test and one-way analysis of variance. *p* values < 0.01(**), and < 0.05(*).

Journal of Cell Science • Supplementary information

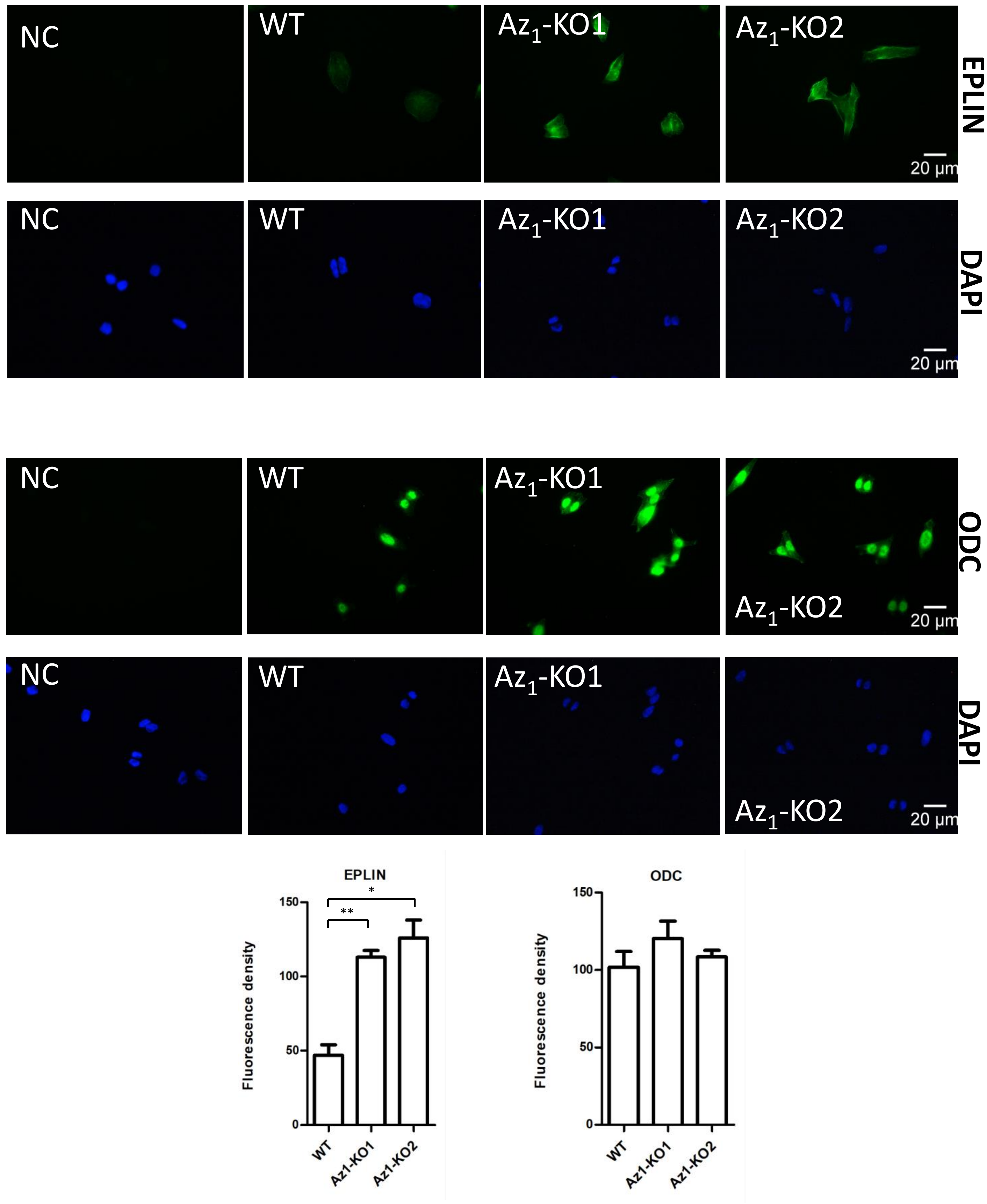

Fig. S2. Expression levels and localization of EPLIN-β and ODC in HCT116 parental (WT) and $Az_1^{-/-}$ (KO) cells

Cells were fixed and stained with mouse anti-EPLIN (top panel) or anti-ODC (middle panel), followed by anti-mouse IgG (green) staining. HCT116 parental cells stained for normal mouse IgG, followed by anti-mouse IgG as negative control (NC). The quantitative results from three images are shown at the bottom panel. Scale bars are labelled at the right panel images of each antibody. HCT116 cells express only EPLIN-β. Statistical comparisons between two groups were carried out by Student's t-test and one-way analysis of variance. p values < 0.01(**), and < 0.05(*).

Blot transparency

Fig. 2C

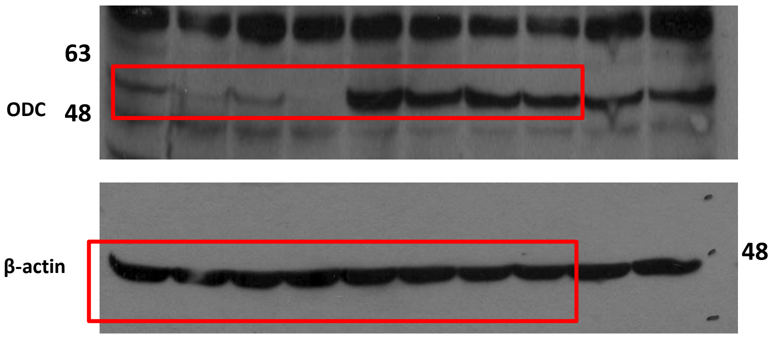

Fig. 2D

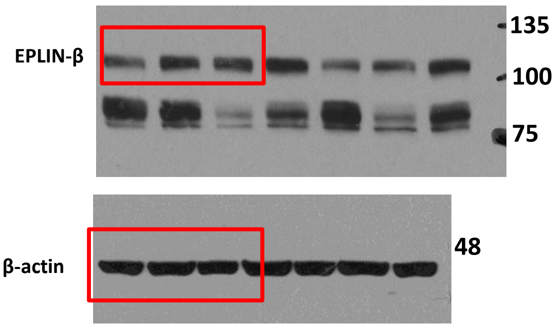

Fig. 2E

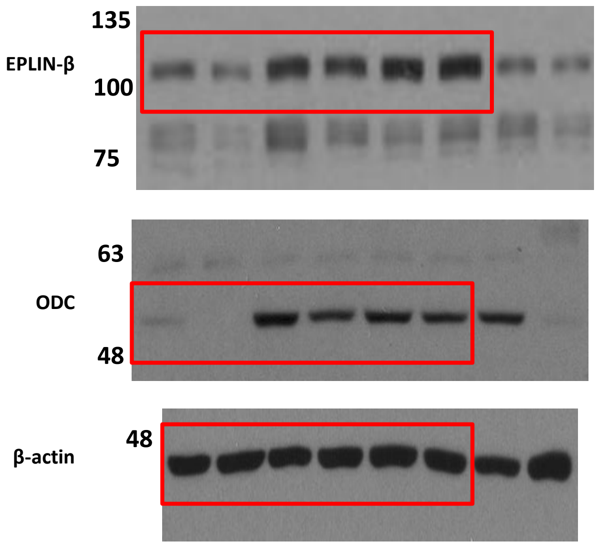

Fig. 2G

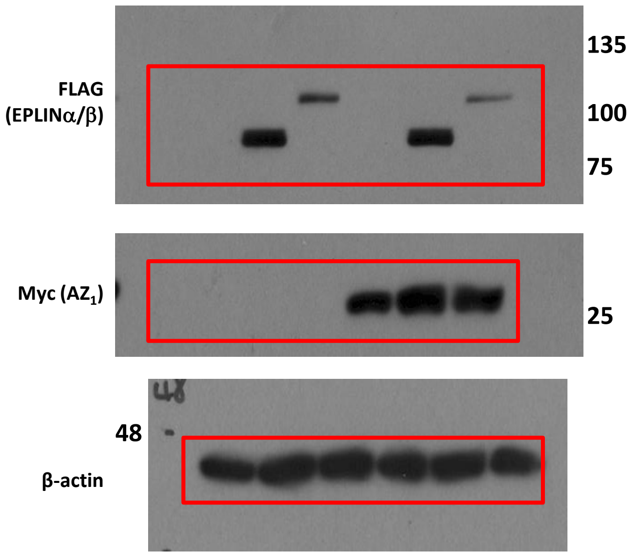

Fig. 2F

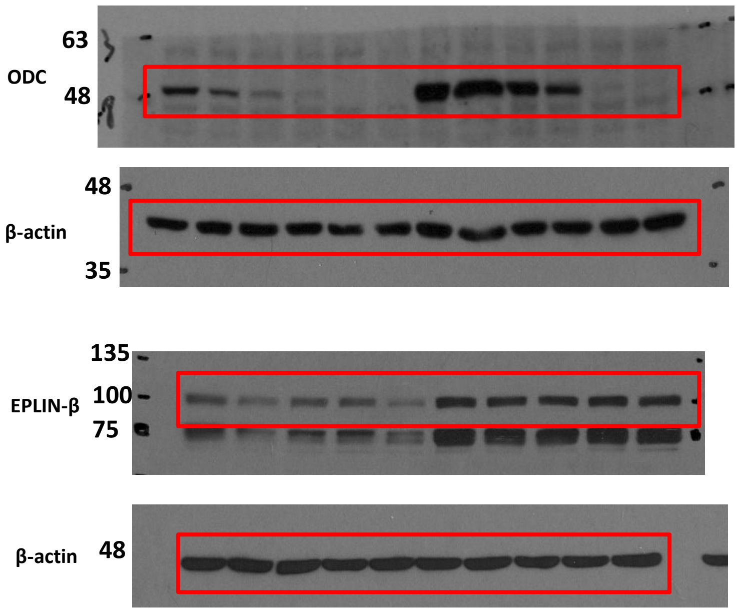

Fig. S3. Blot transparency for the blots used in Fig. 2

All primary data used in Fig. 2 are presented here as uncropped images, and the bands used are shown in red boxes.

C

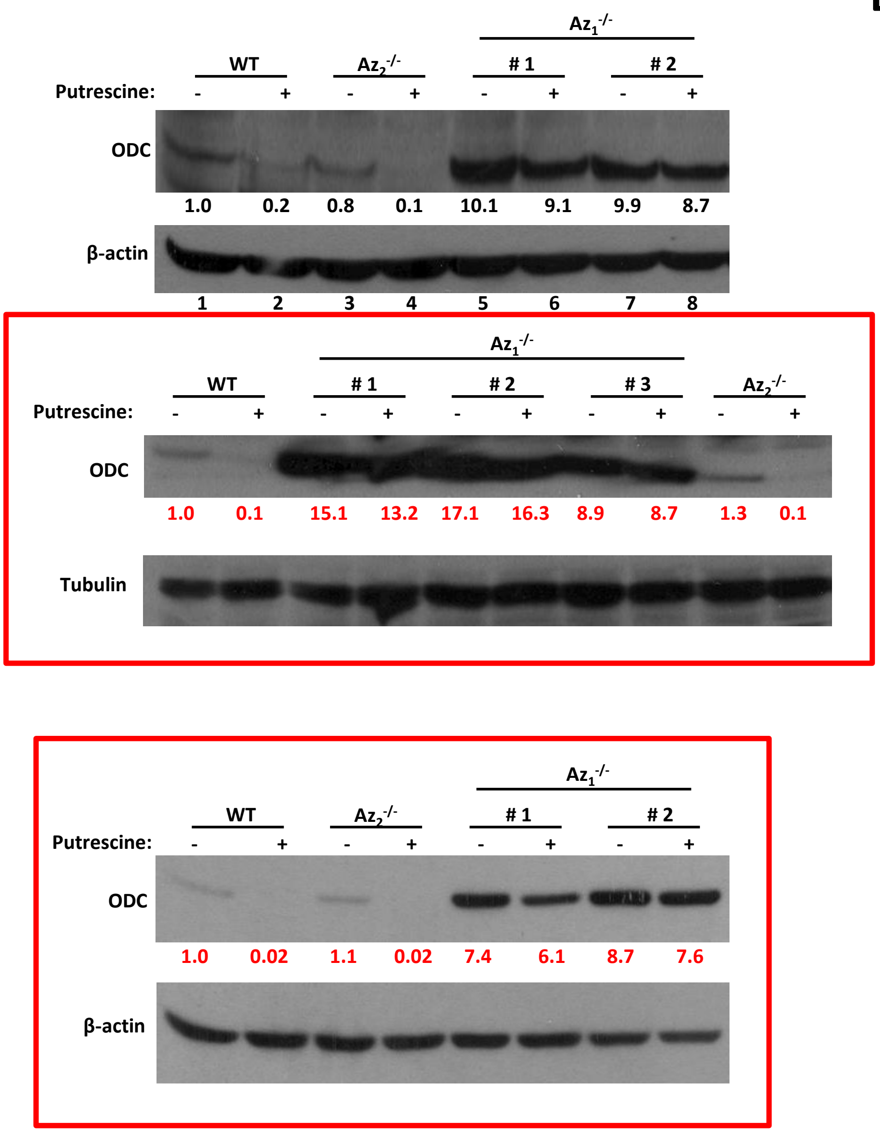

D

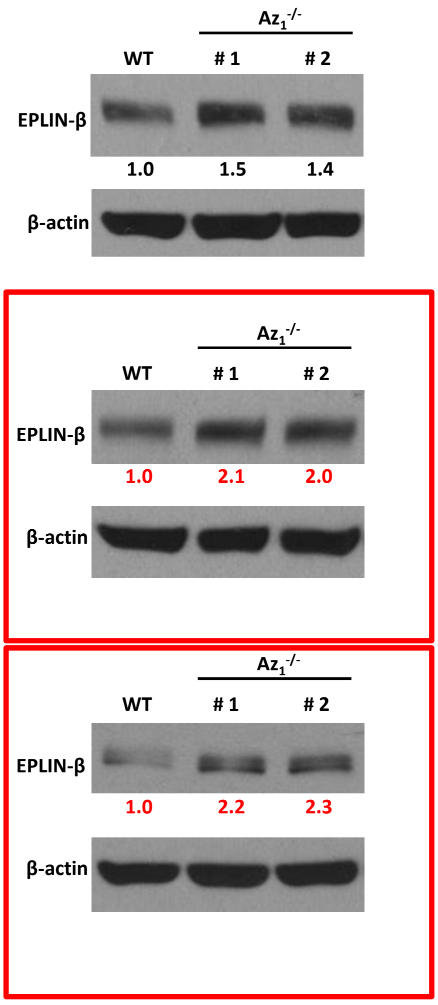

Fig. S4. Repeated blots for Fig. 2

Journal of Cell Science • Supplementary information

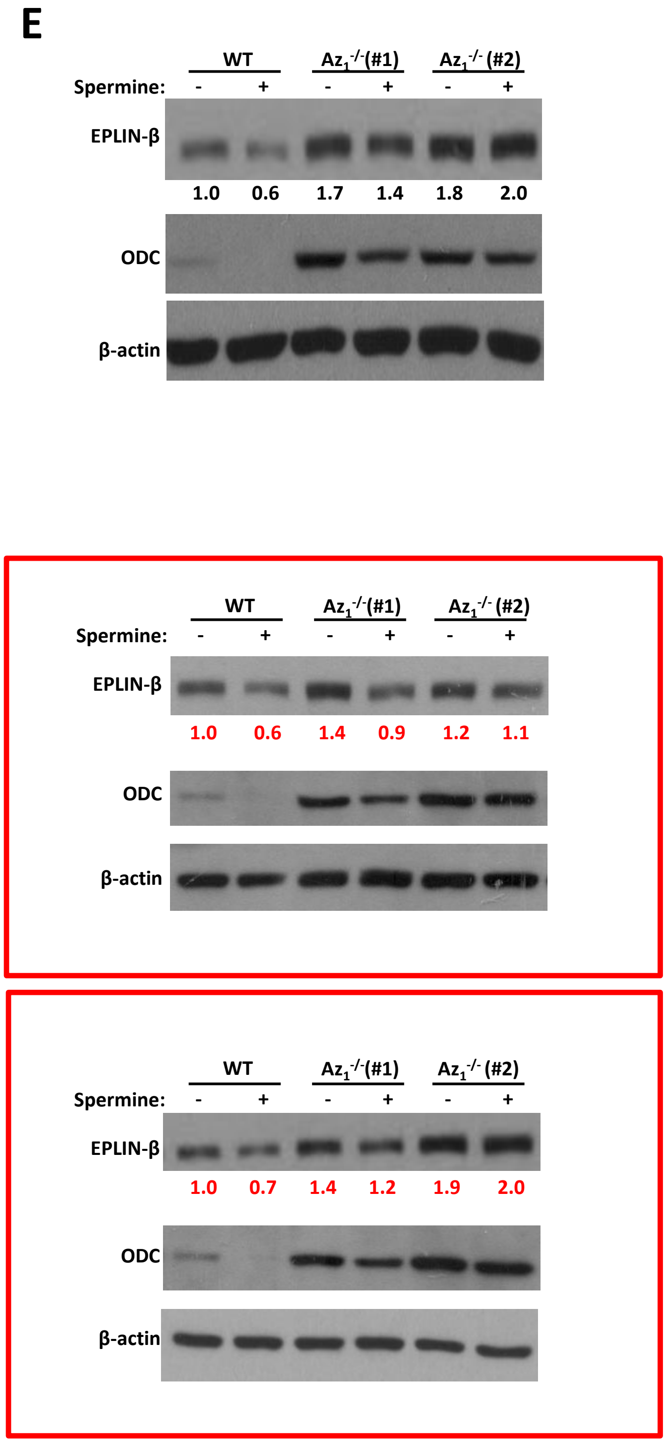

Fig. S4. Repeated blots for Fig. 2

Journal of Cell Science • Supplementary information

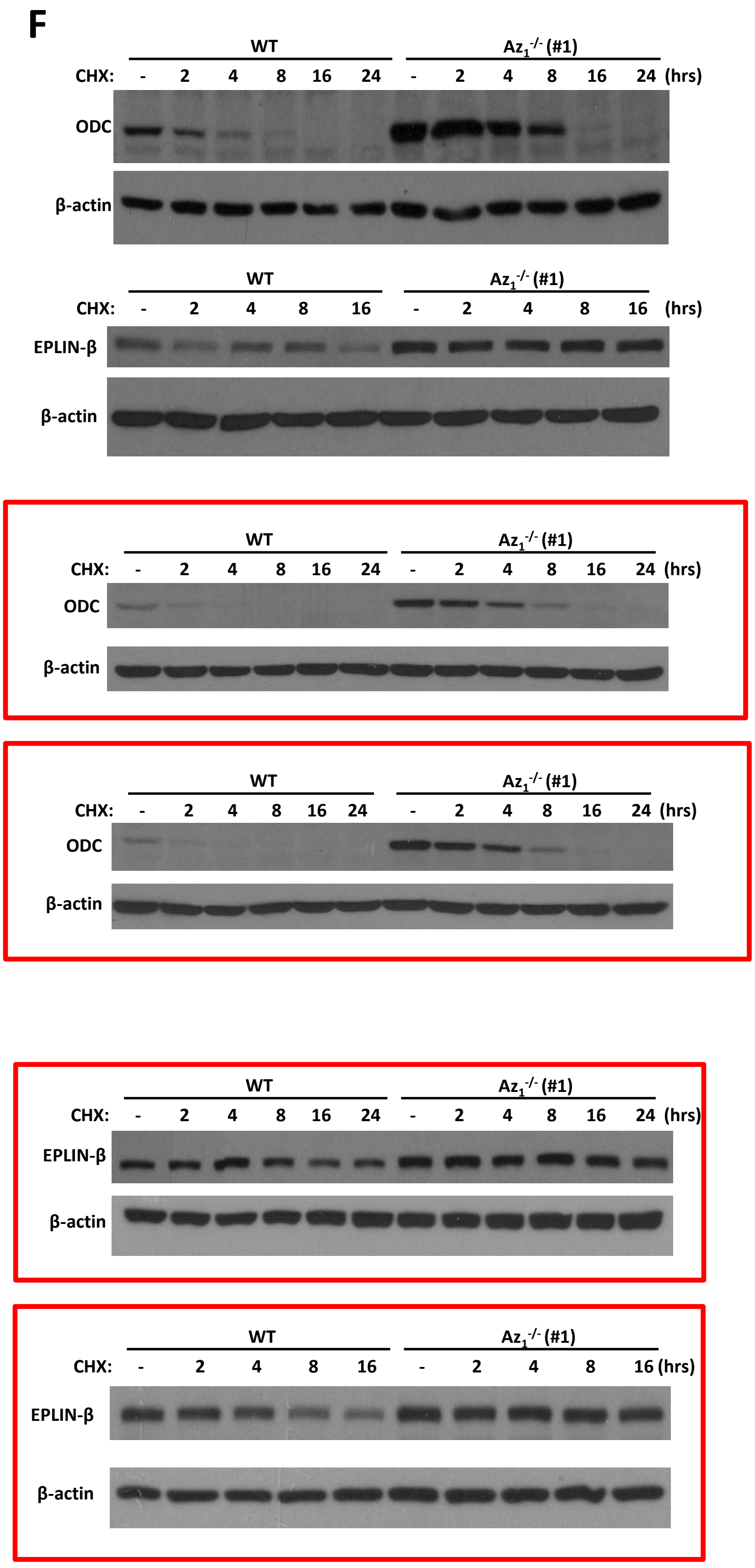

Fig. S4. Repeated blots for Fig. 2

Journal of Cell Science • Supplementary information

G

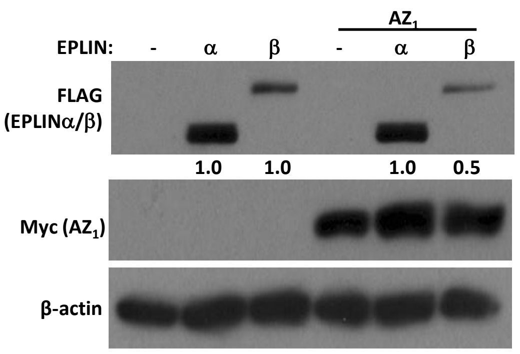

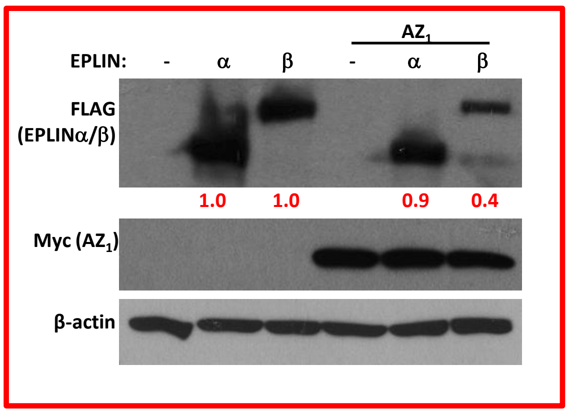

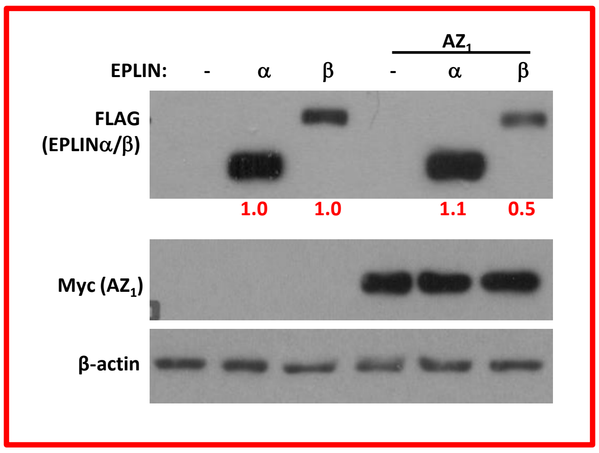

Fig. S4. Repeated blots for Fig. 2

Journal of Cell Science • Supplementary information

Fig. 2C

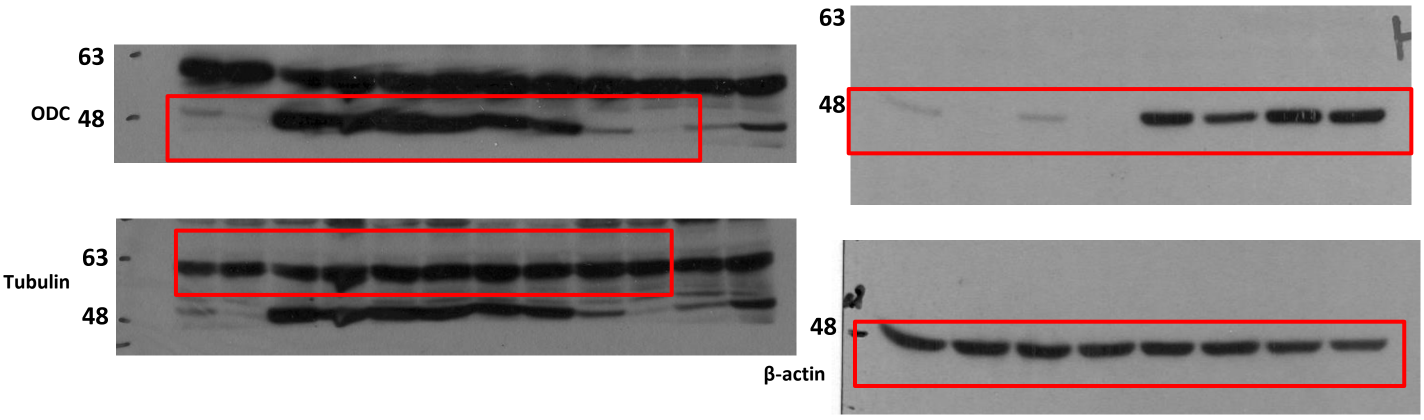

Fig. 2D

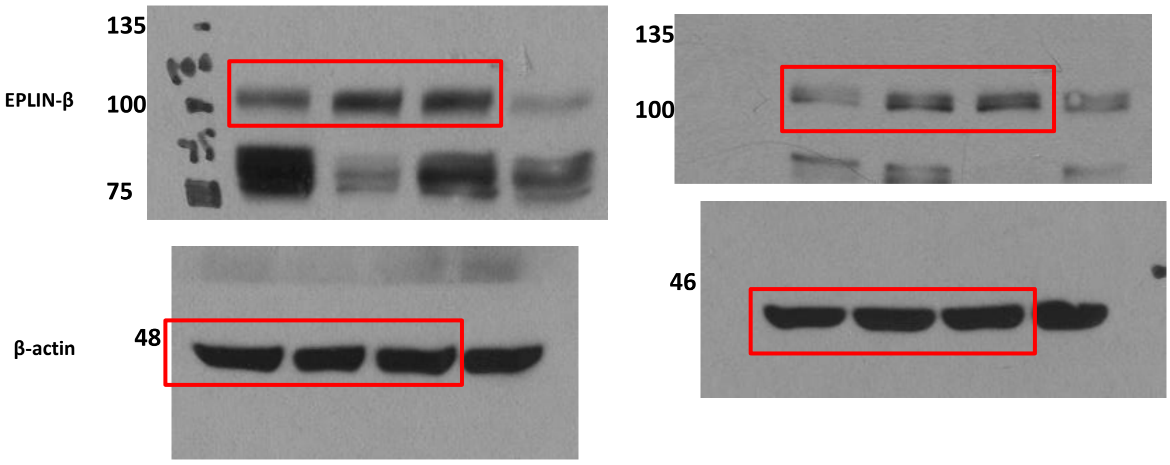

Fig. 2E

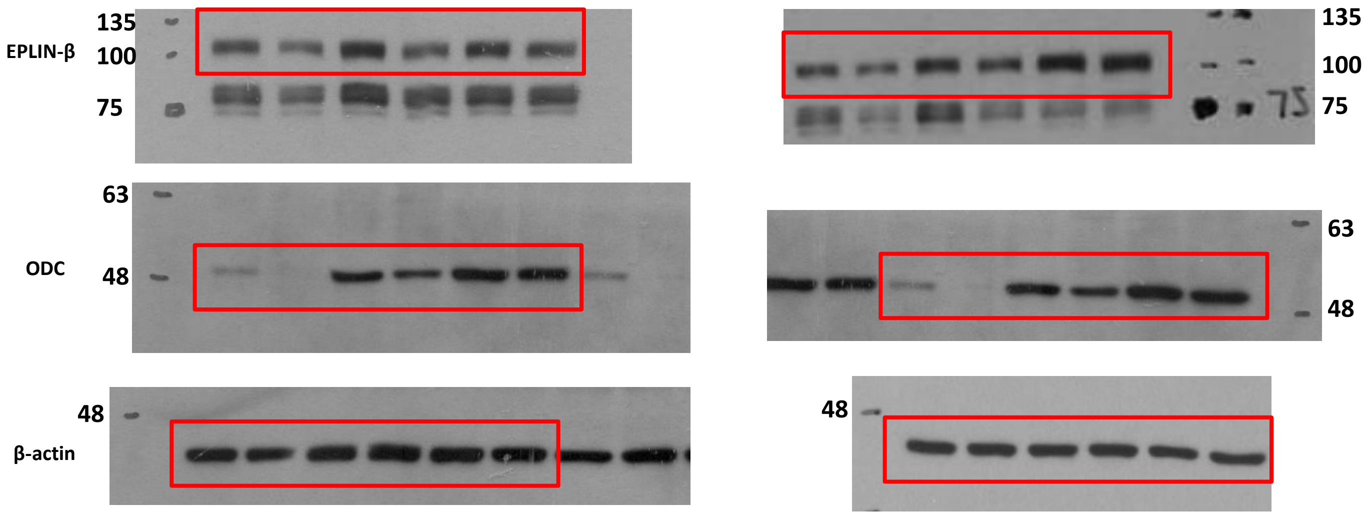

Fig. S5. Blot transparency for the repeated blots of Fig. 2

Journal of Cell Science • Supplementary information

Fig. 2F

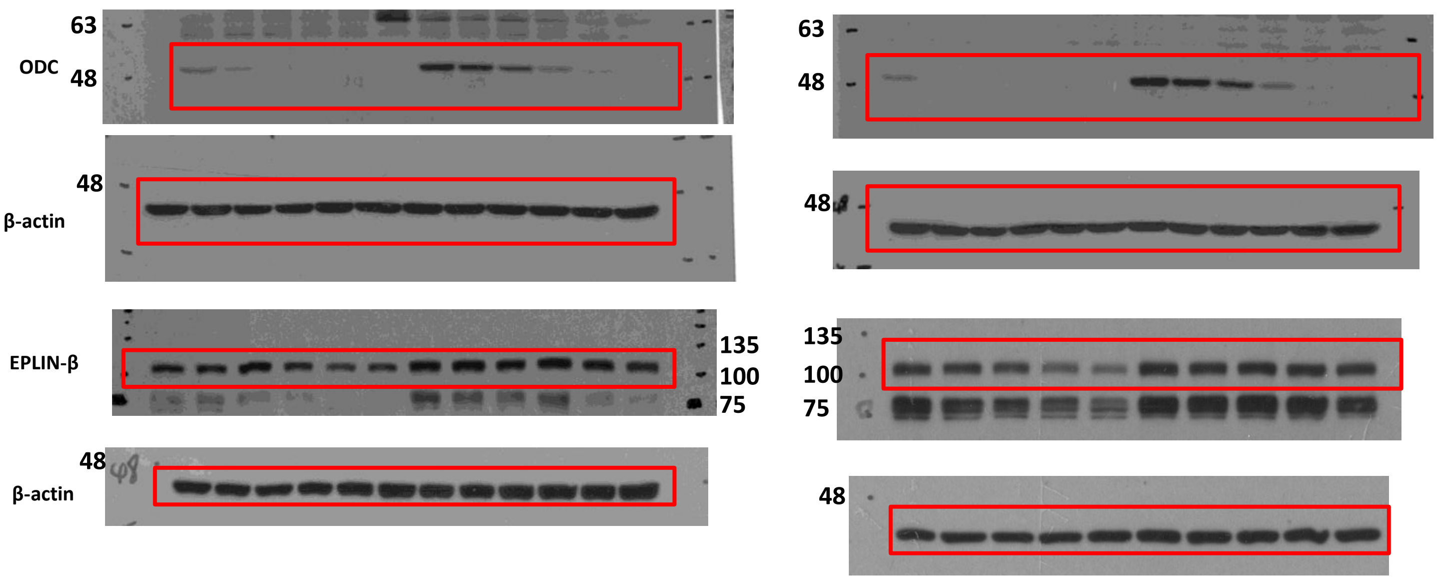

Fig. 2G

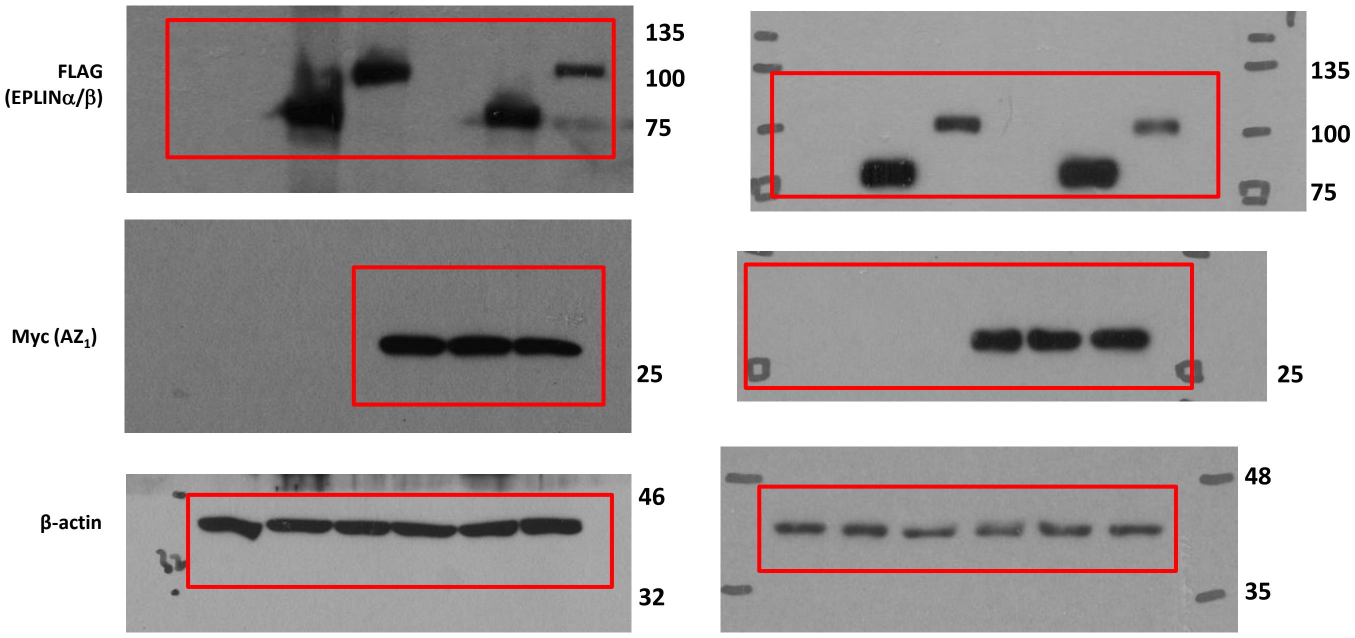

Fig. S5. Blot transparency for the repeated blots of Fig. 2

All repeated blots (for Fig. 2) are presented in the red boxes, along with uncropped images (for blot transparency).

Journal of Cell Science • Supplementary information

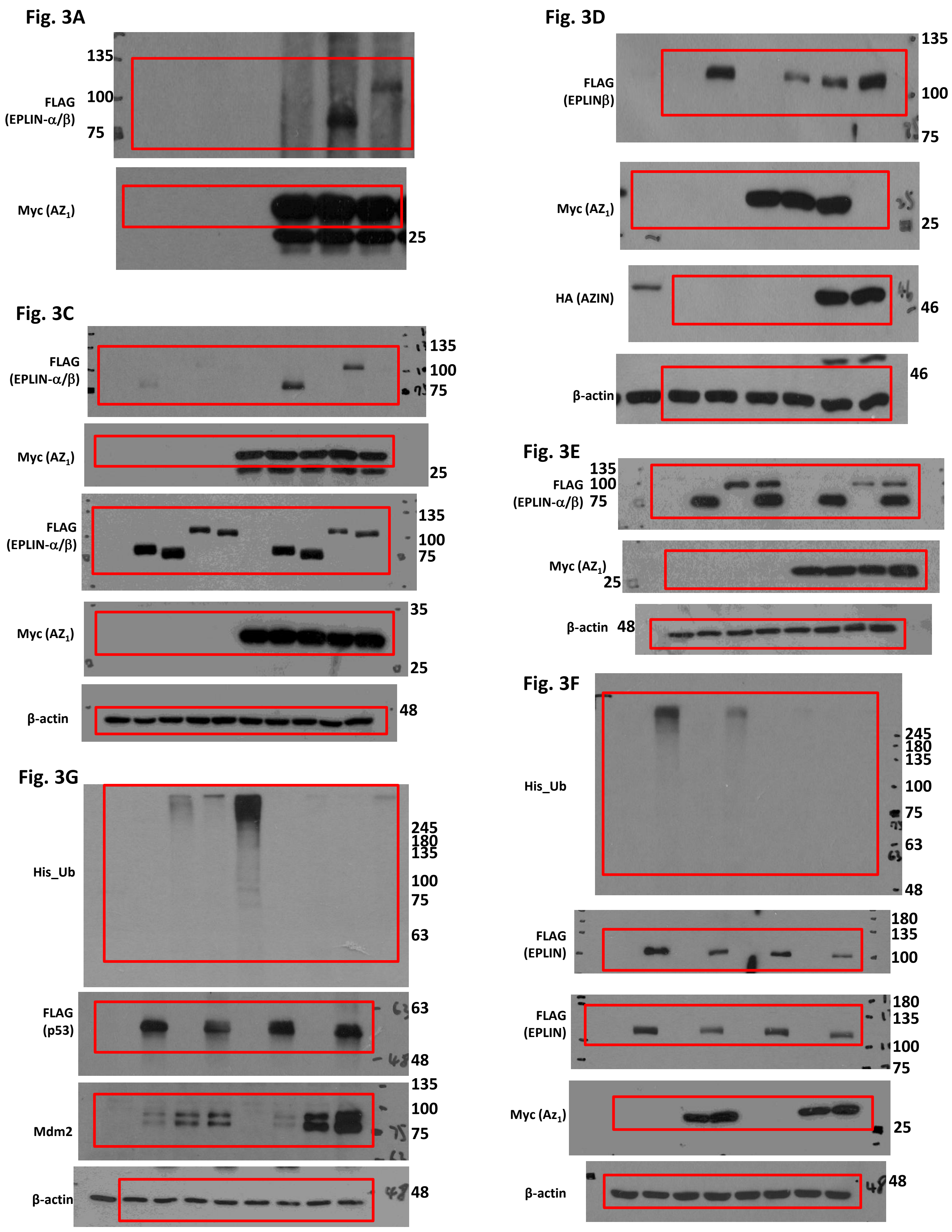

Fig. S6. Blot transparency for the blots used in Fig. 3
All primary data used in Fig. 3 are presented here as uncropped images, and the bands used are shown in red boxes.

Journal of Cell Science • Supplementary information

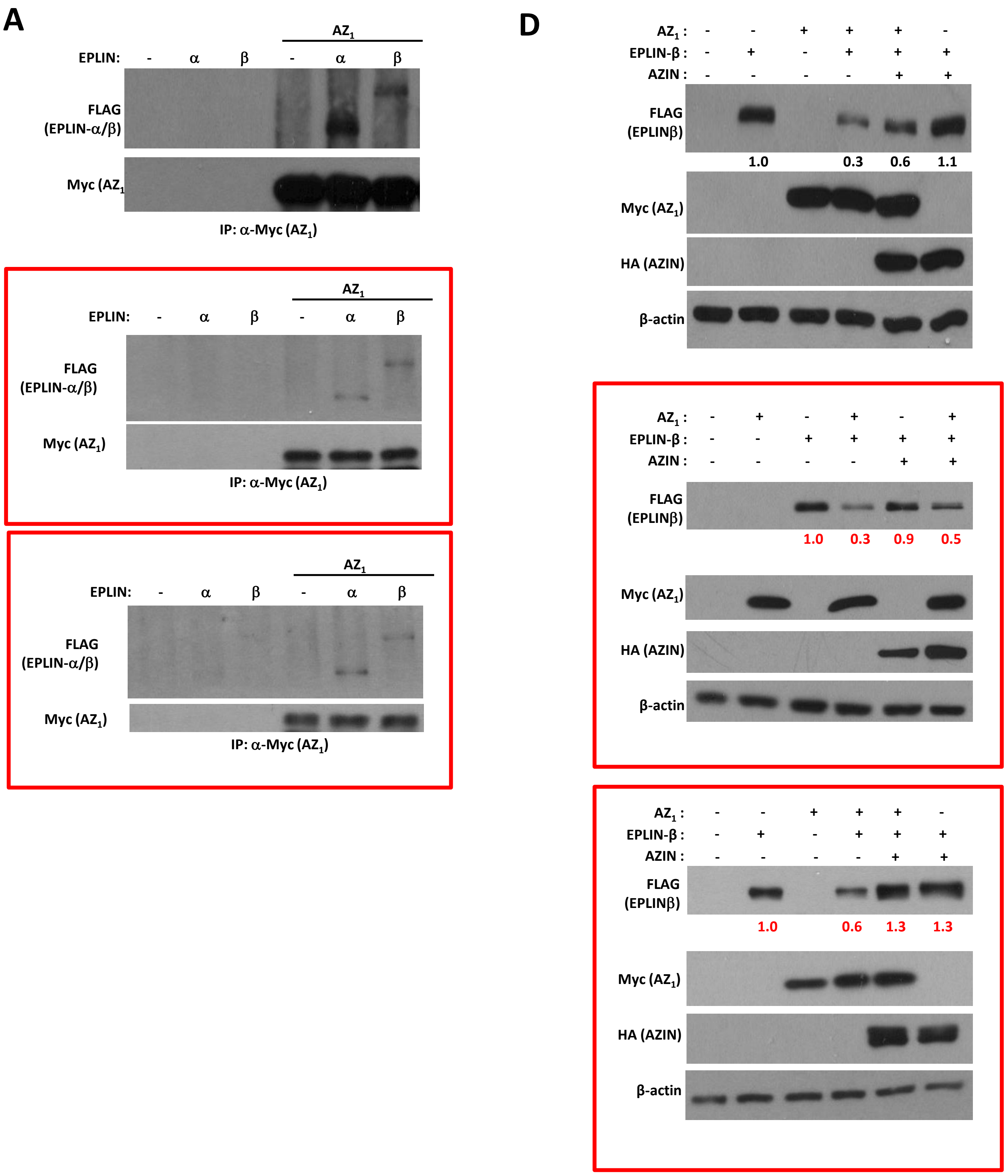

Fig. S7. Repeated blots for Fig. 3

Journal of Cell Science • Supplementary information

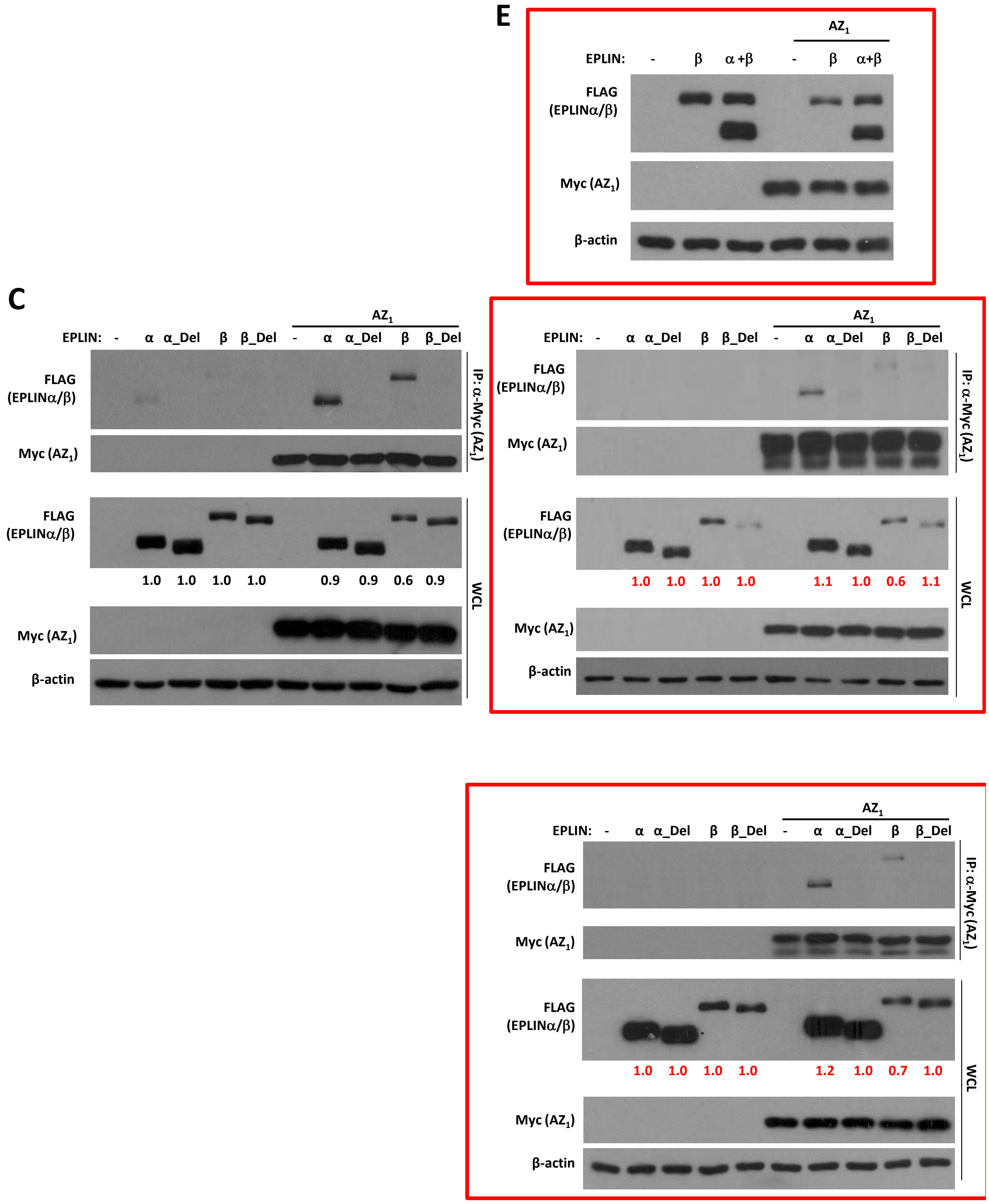

Fig. S7. Repeated blots for Fig. 3

Journal of Cell Science • Supplementary information

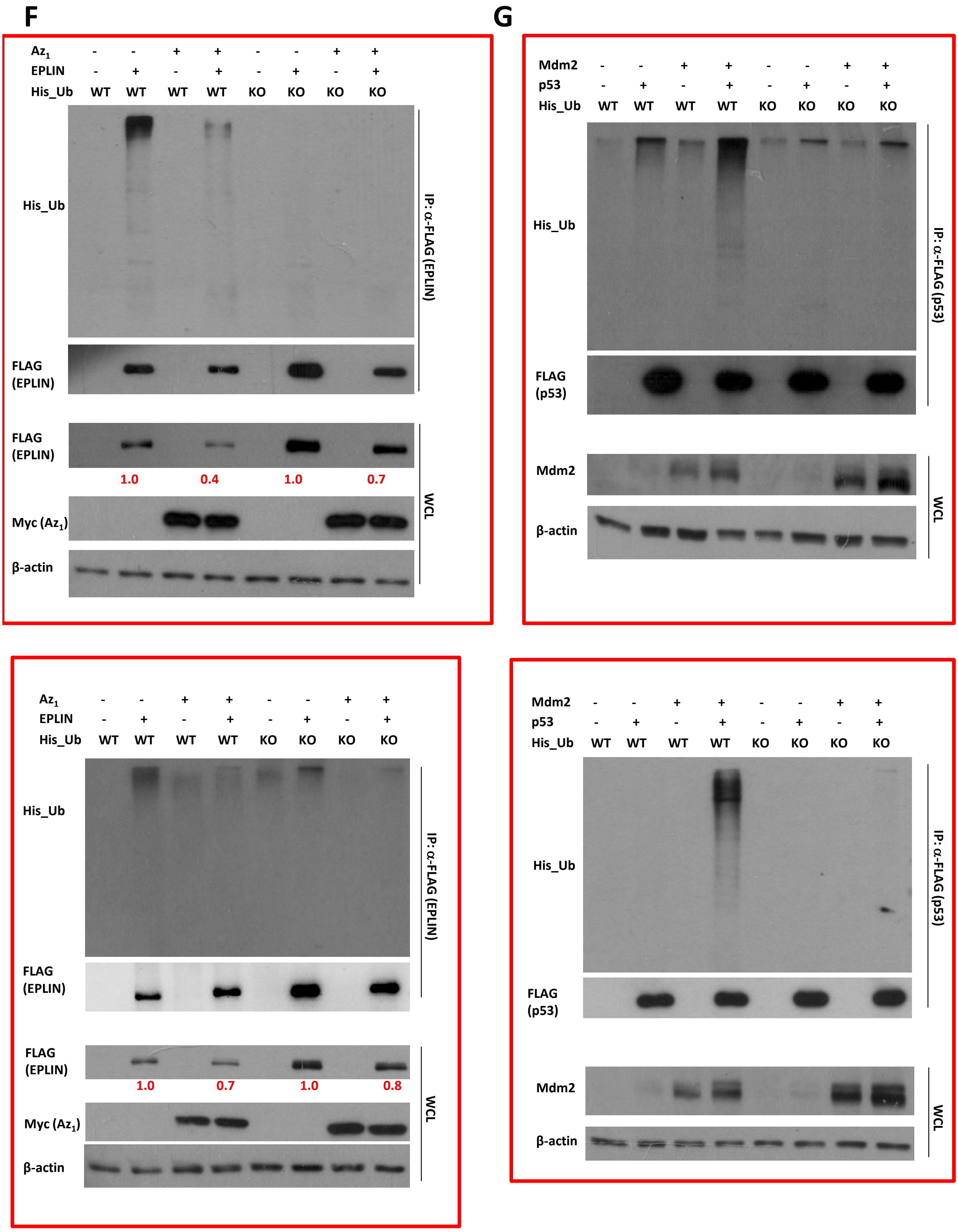

Fig. S7. Repeated blots for Fig. 3

Journal of Cell Science • Supplementary information

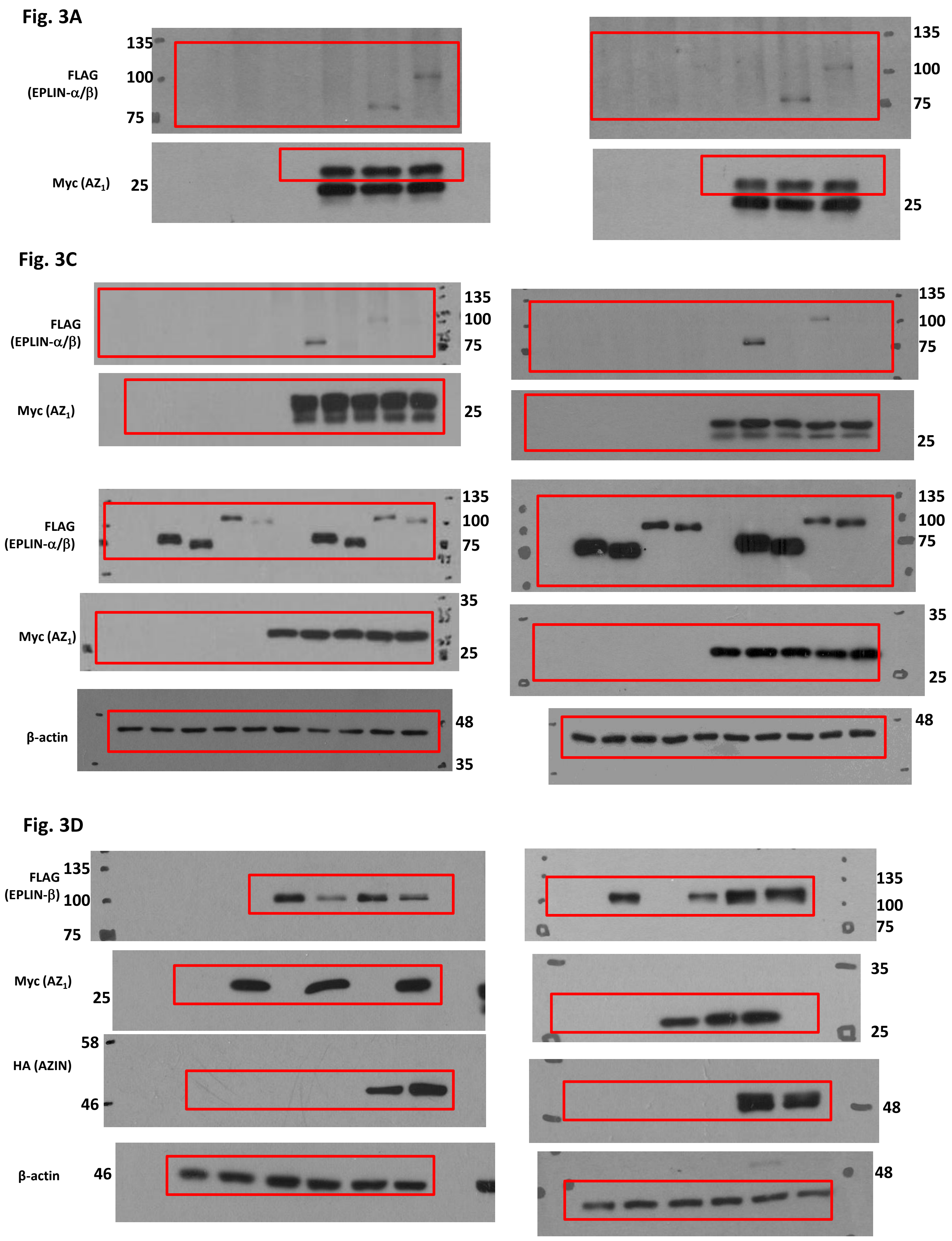

Fig. S8. Blot transparency for the repeated blots of Fig. 3

Journal of Cell Science • Supplementary information

Fig. 3F

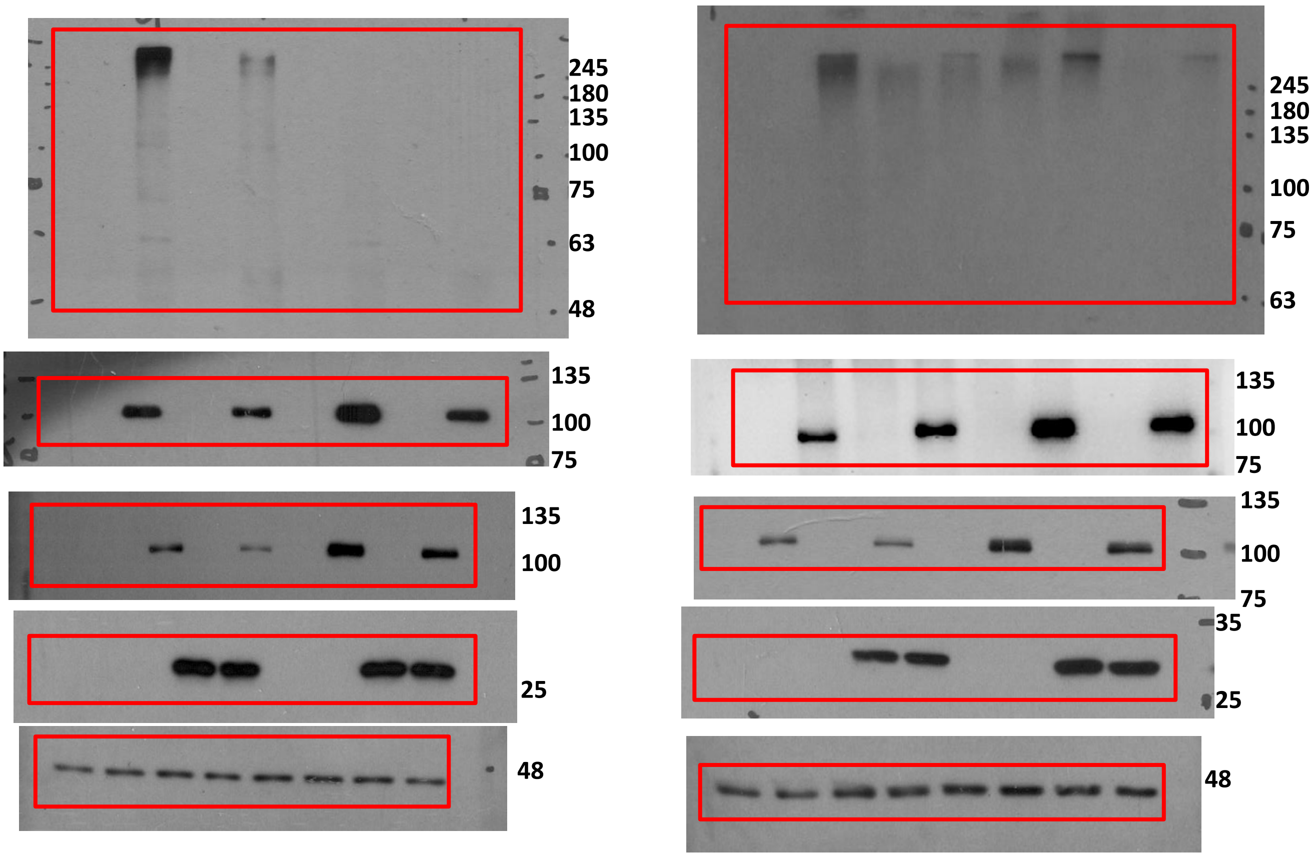

Fig. 3G

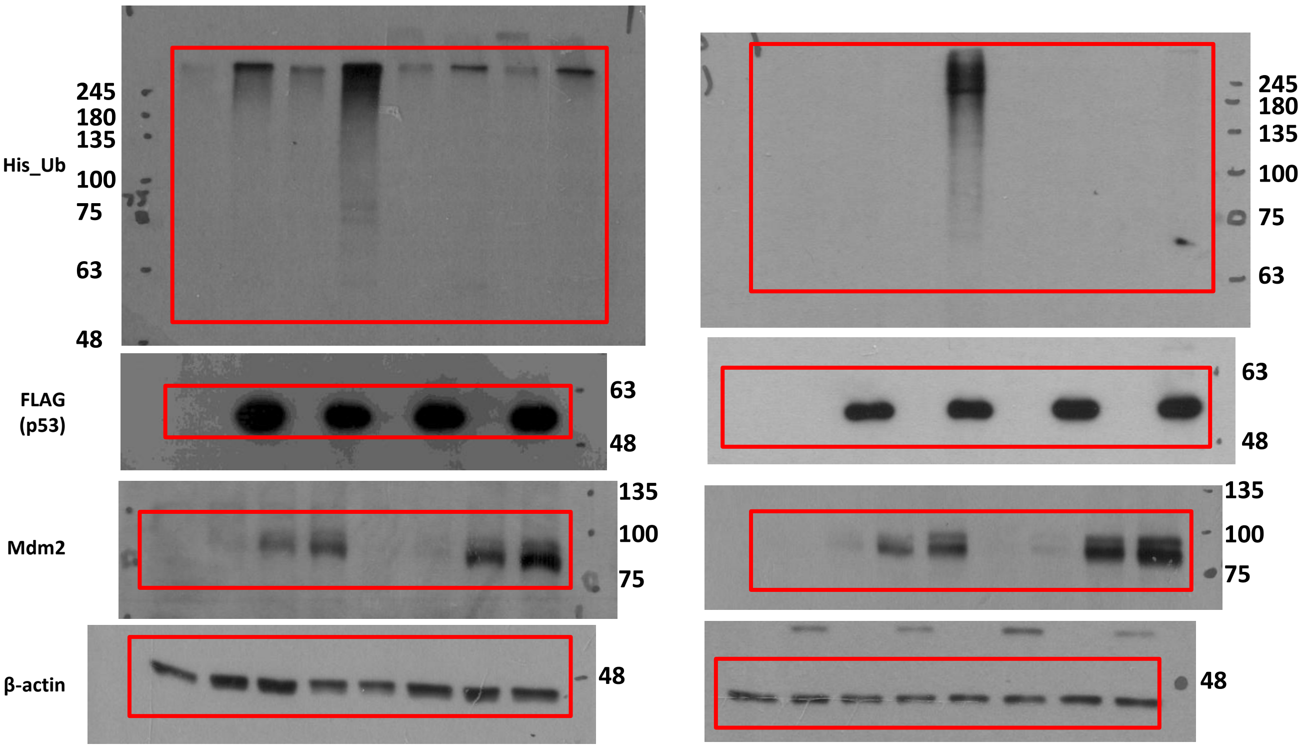

Fig. S8. Blot transparency for the repeated blots of Fig. 3

Journal of Cell Science • Supplementary information

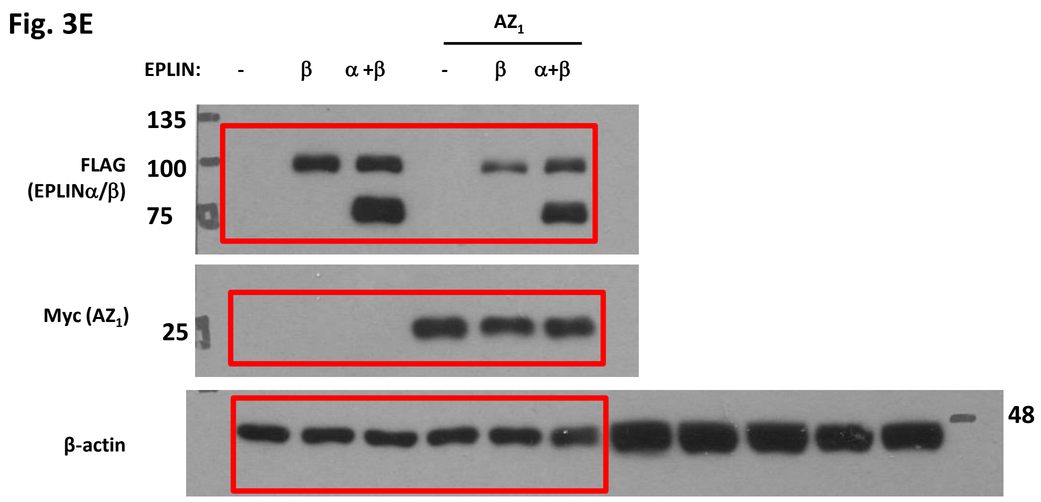

Fig. S8. Blot transparency for the repeated blots of Fig. 3
All repeated blots (for Fig. 3) are presented in the red boxes, along with uncropped images (for blot transparency).

Fig. 4C

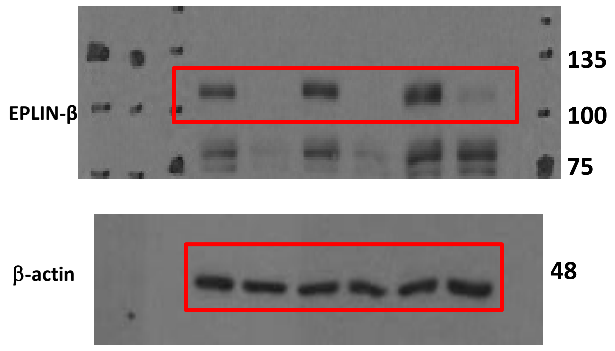

Fig. S1A

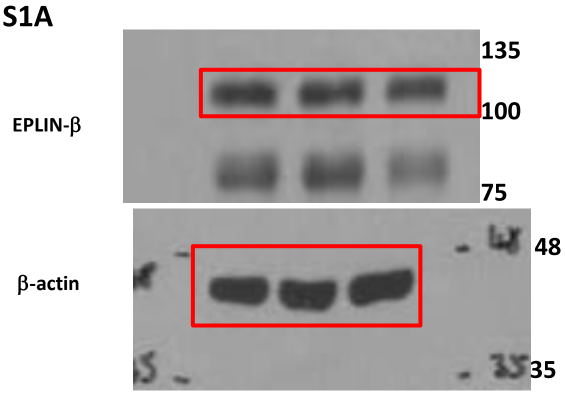

Fig. S1C

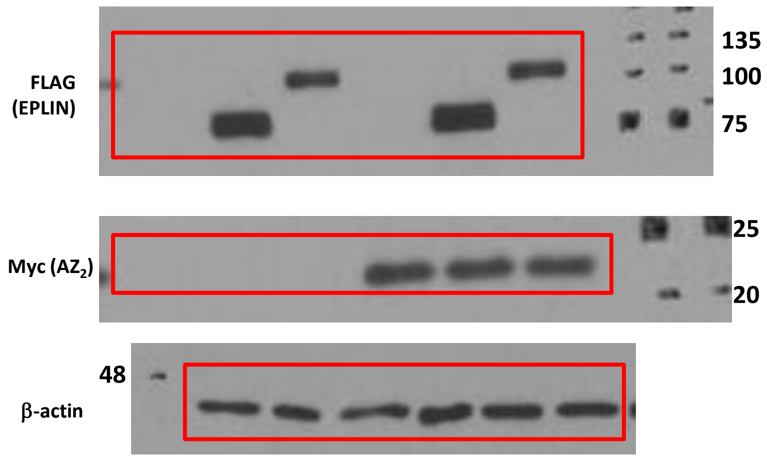

Fig. S9. Blot transparency for the blots or images used in Fig. 4 and Fig. S1
All primary data used in Fig. 4 and Fig. S1 are presented here as uncropped images, and the bands used are shown in red boxes.

Journal of Cell Science • Supplementary information

Fig. 4C

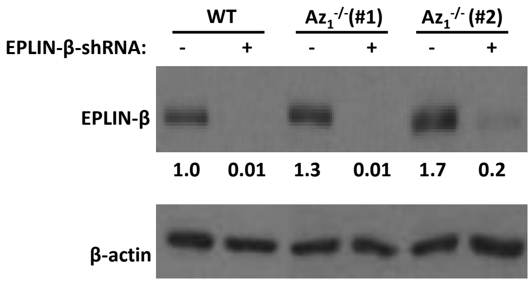

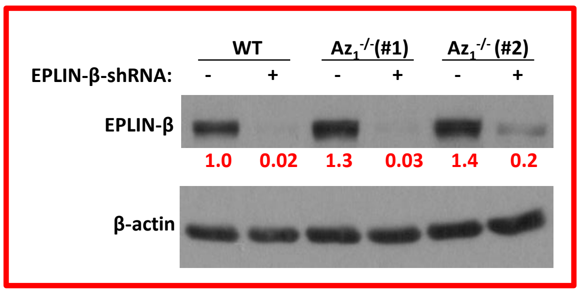

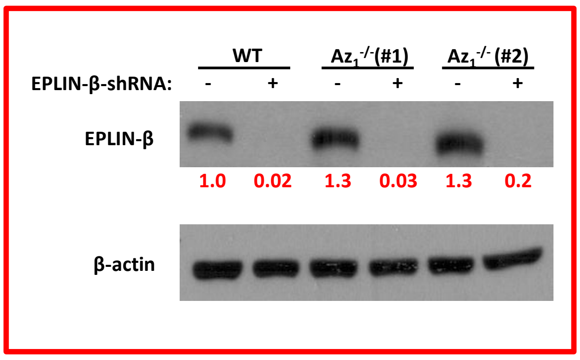

Fig. S10. Repeated blots or images for Fig. 4 and Fig. S1

Journal of Cell Science • Supplementary information

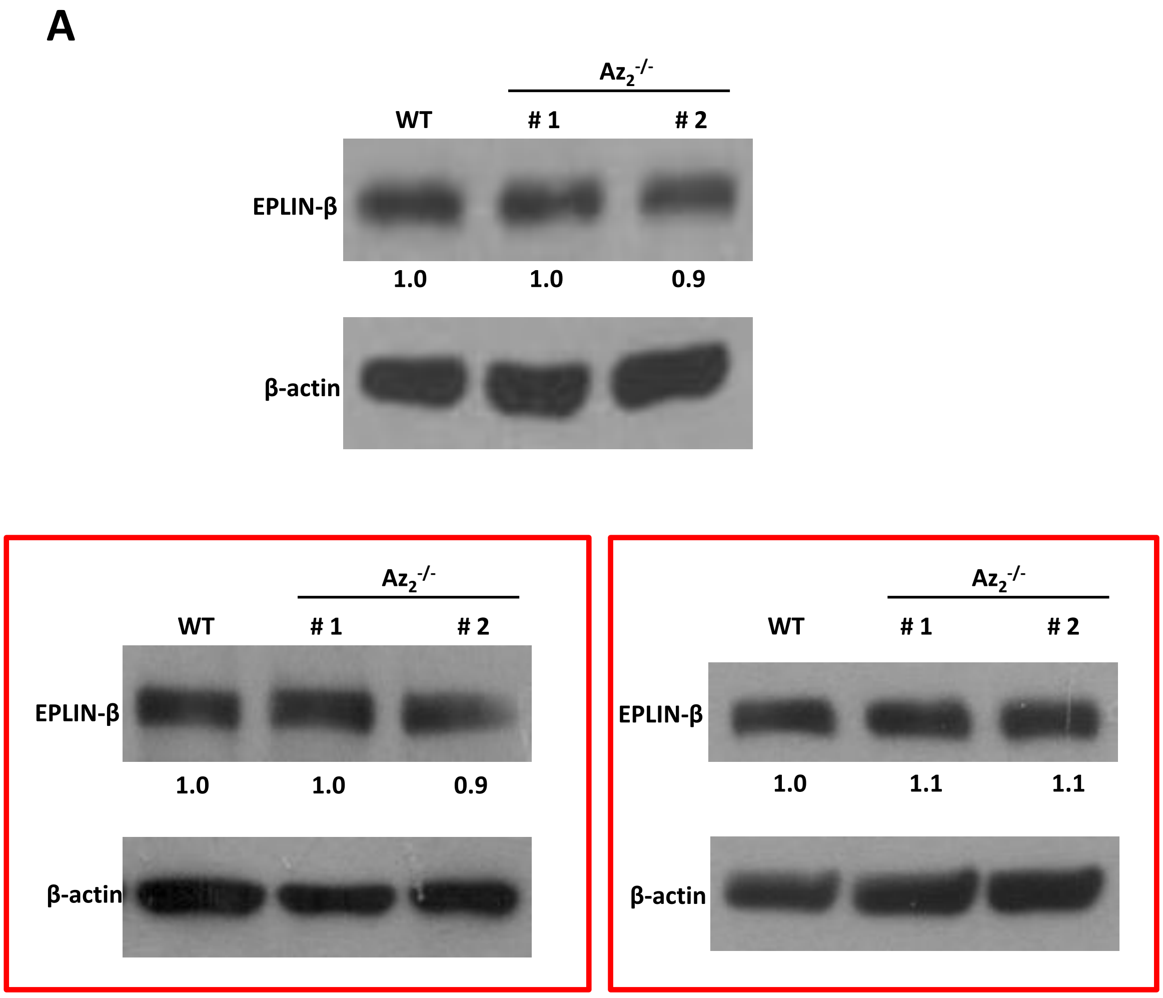

Fig. S10. Repeated blots or images for Fig. 4 and Fig. S1

Journal of Cell Science • Supplementary information

C

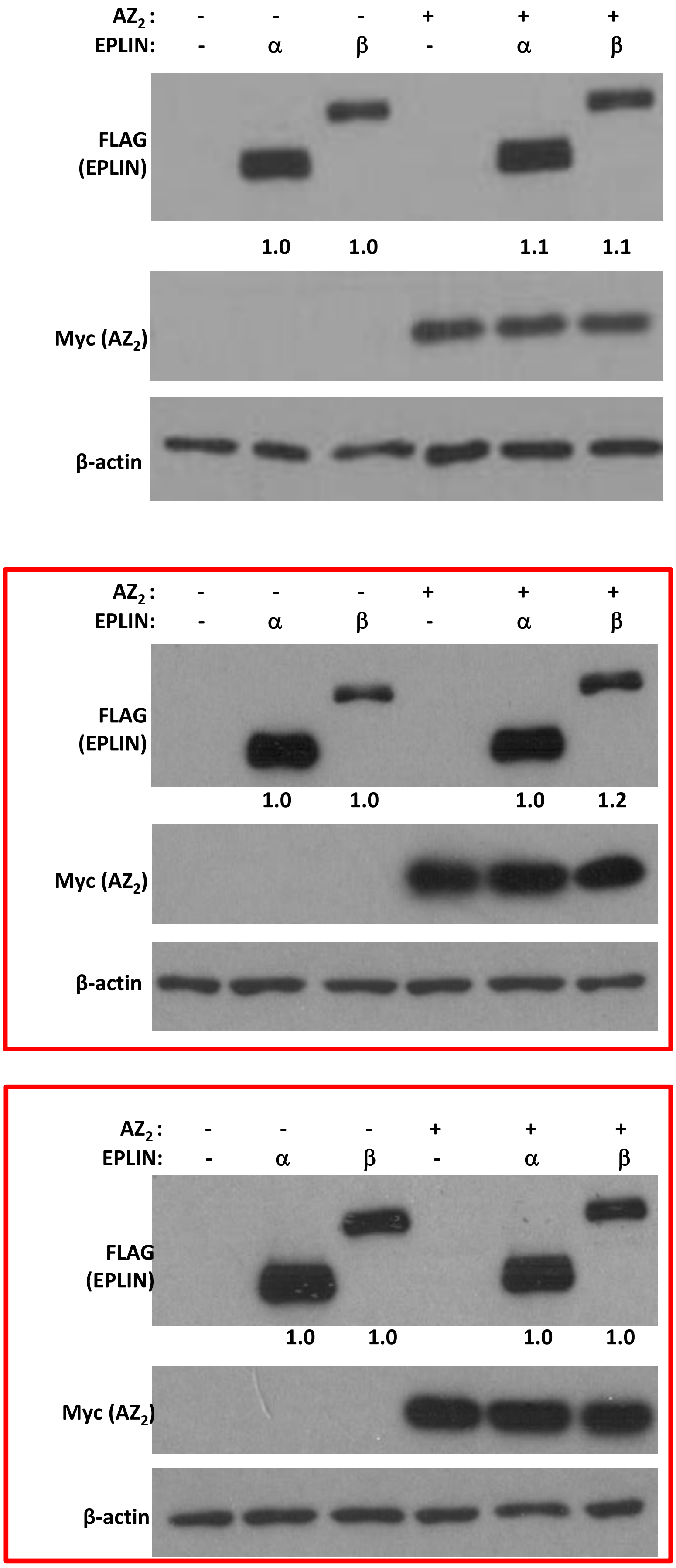

Fig. S10. Repeated blots or images for Fig. 4 and Fig. S1

Journal of Cell Science • Supplementary information

Fig. 4C

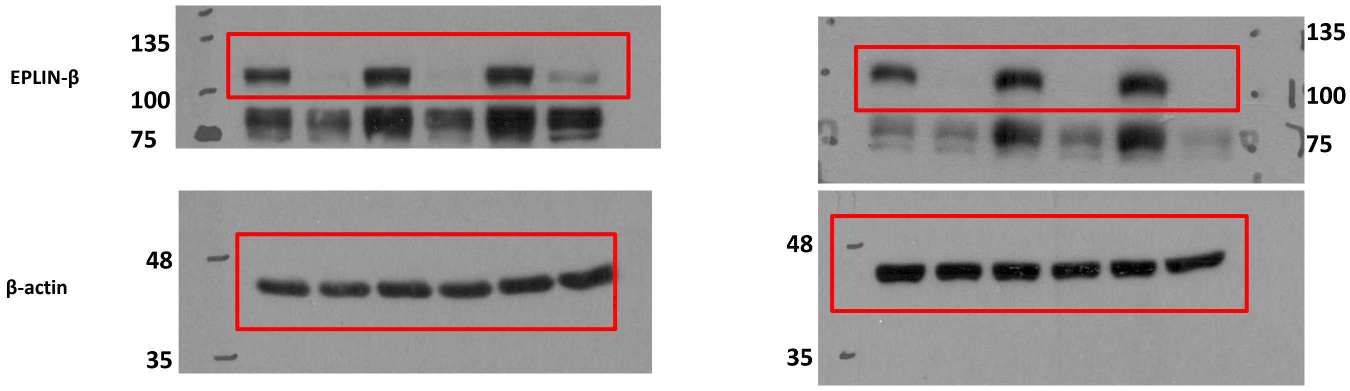

Fig. S11. Blot transparency for repeated blots of Fig. 4 and Fig. S1.

Fig. S1A

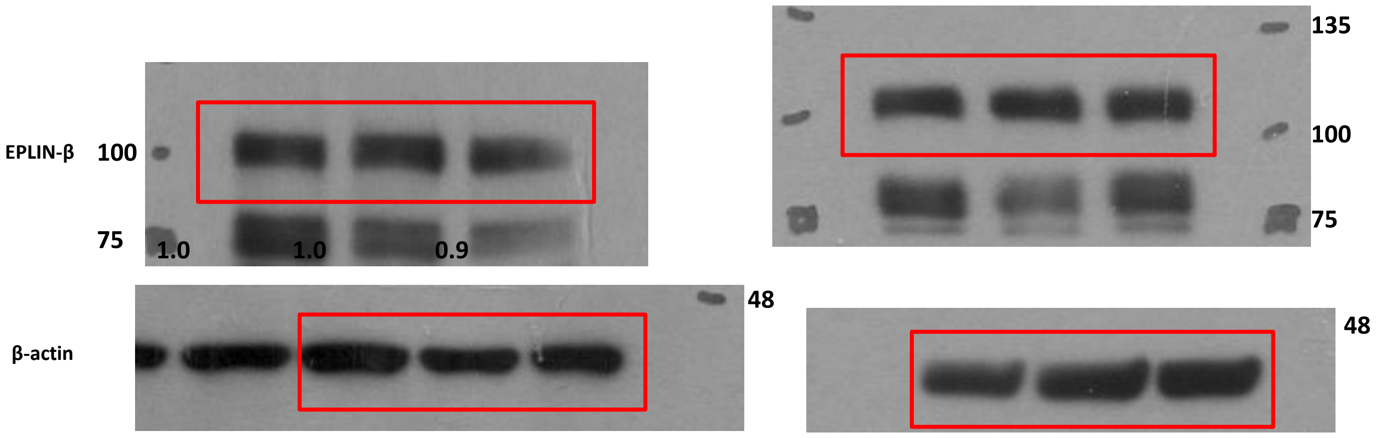

Fig. S1C

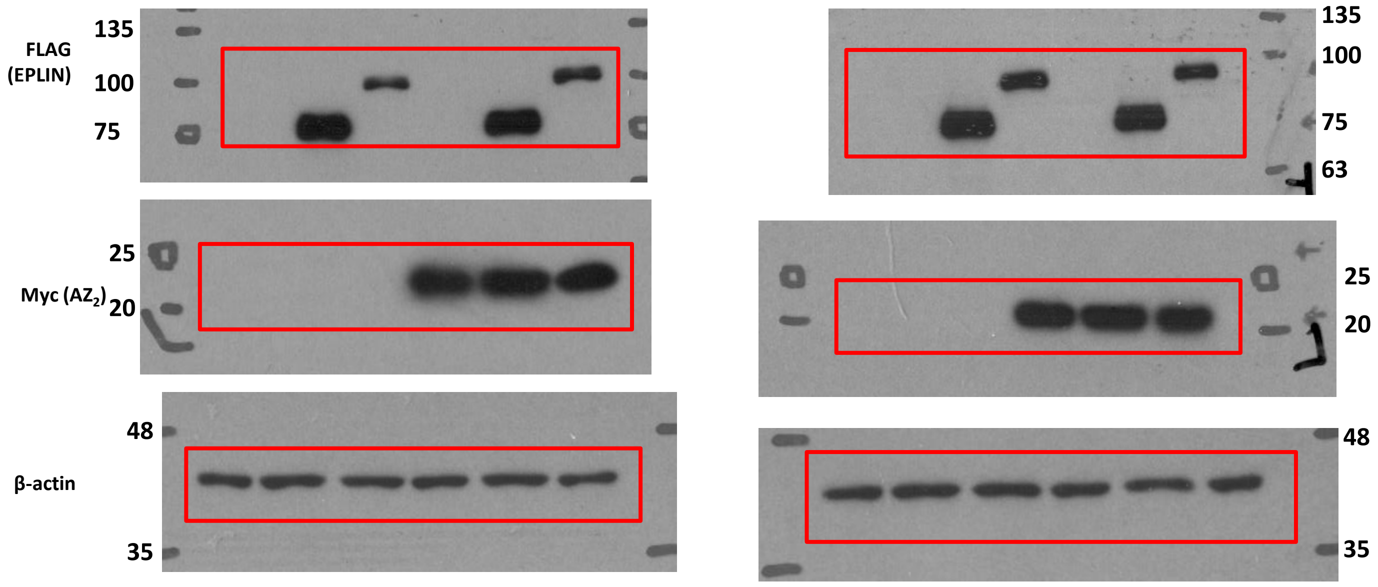

Fig. S11. Blot transparency for repeated blots of Fig. 4 and Fig. S1
All repeated blots for Fig. 4 and Fig. S1 are presented in red boxes, along with the uncropped images (for blot transparency).

Journal of Cell Science • Supplementary information

B

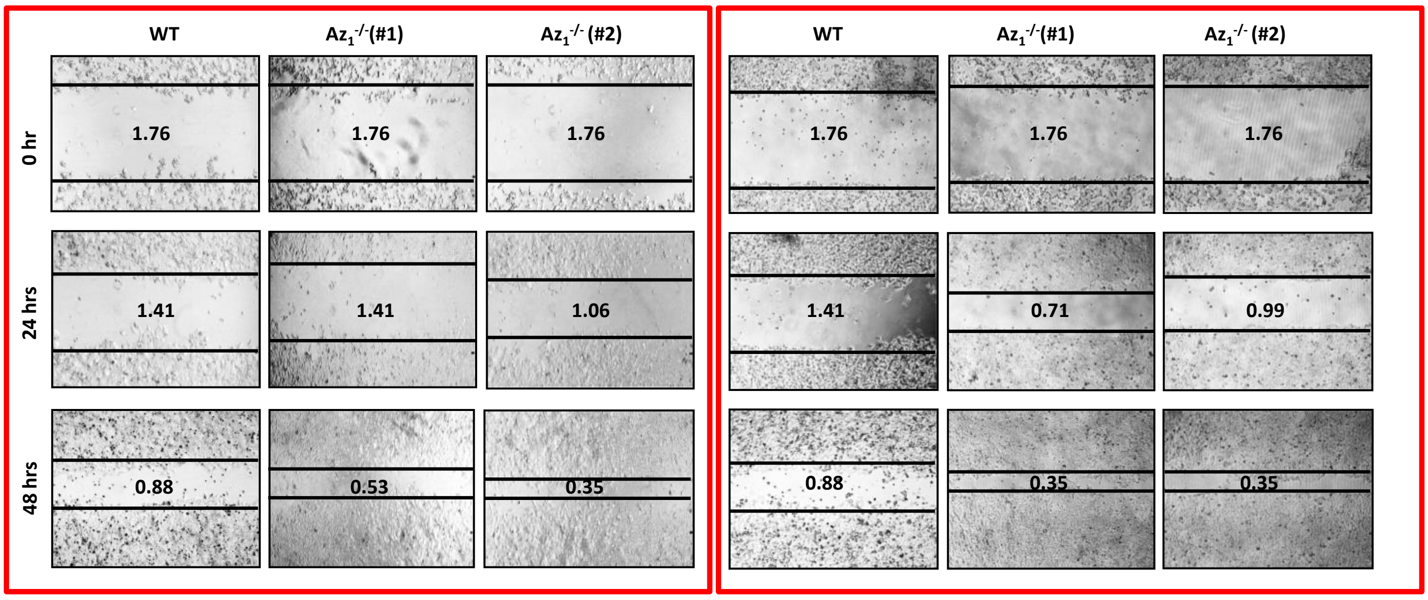

E

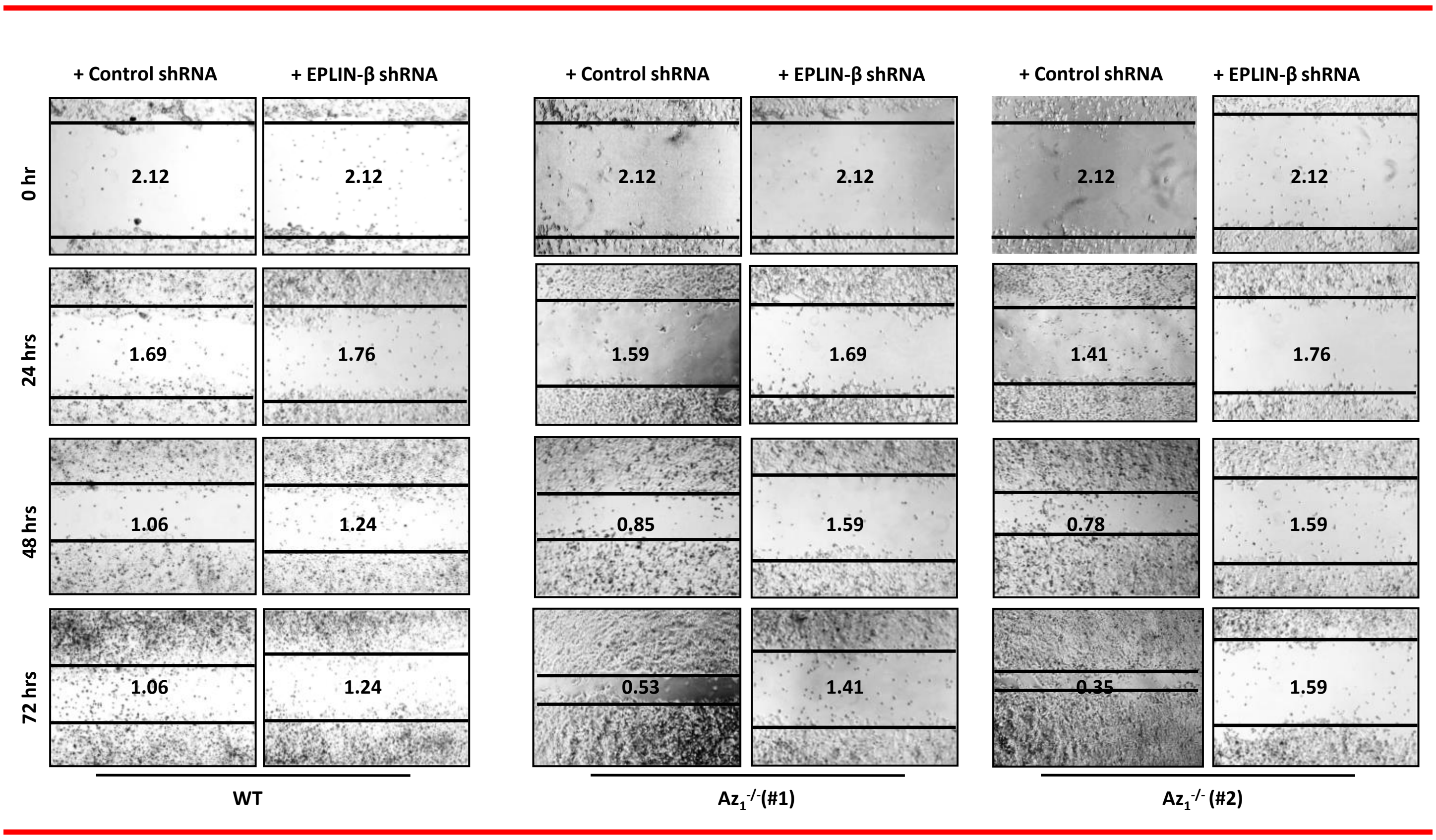

Fig. S12. Repeated images for Fig. 4

Journal of Cell Science • Supplementary information

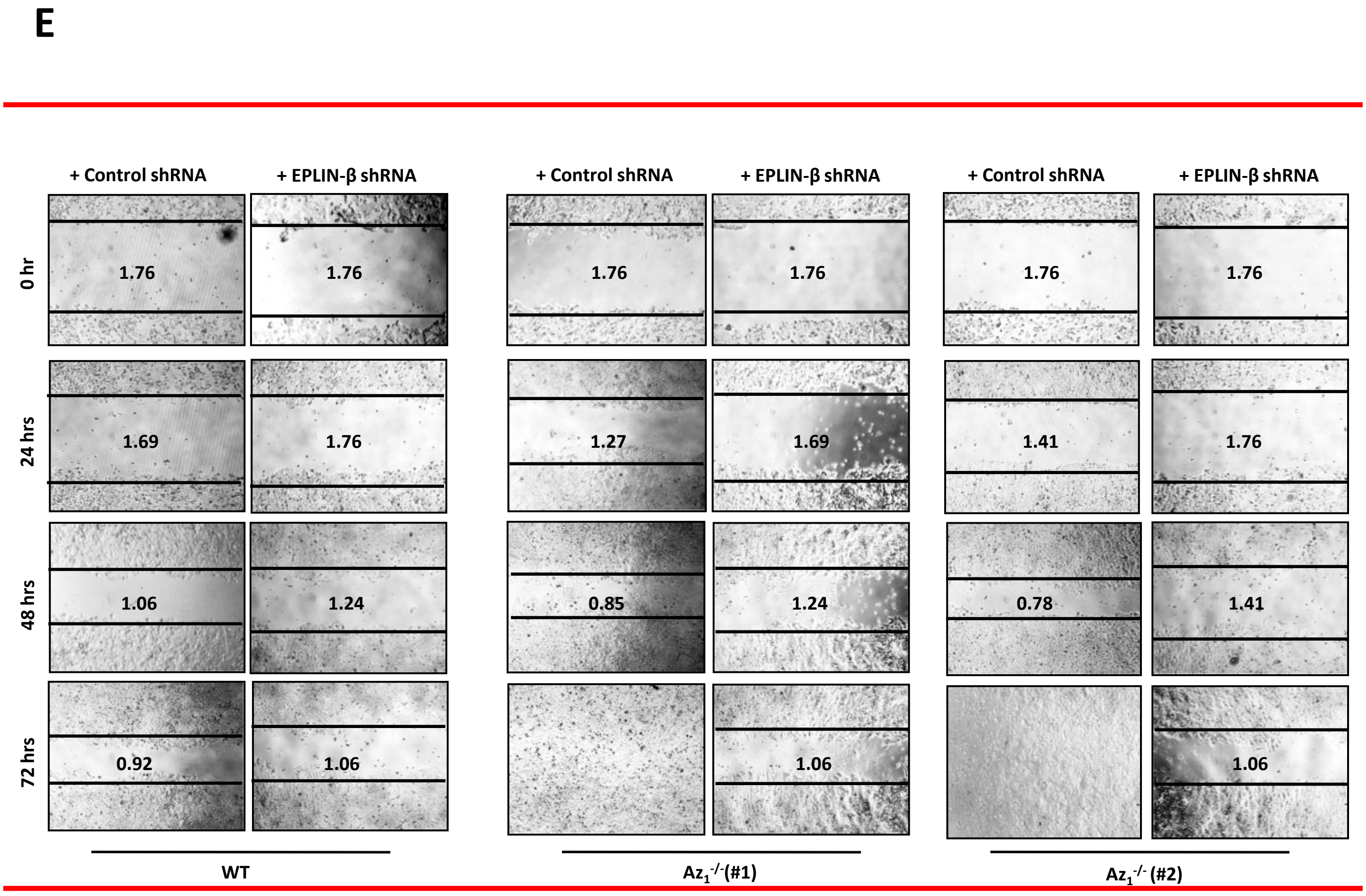

Fig. S12. Repeated images for Fig. 4

Repeated images for Fig. 4 are presented in the red boxes.

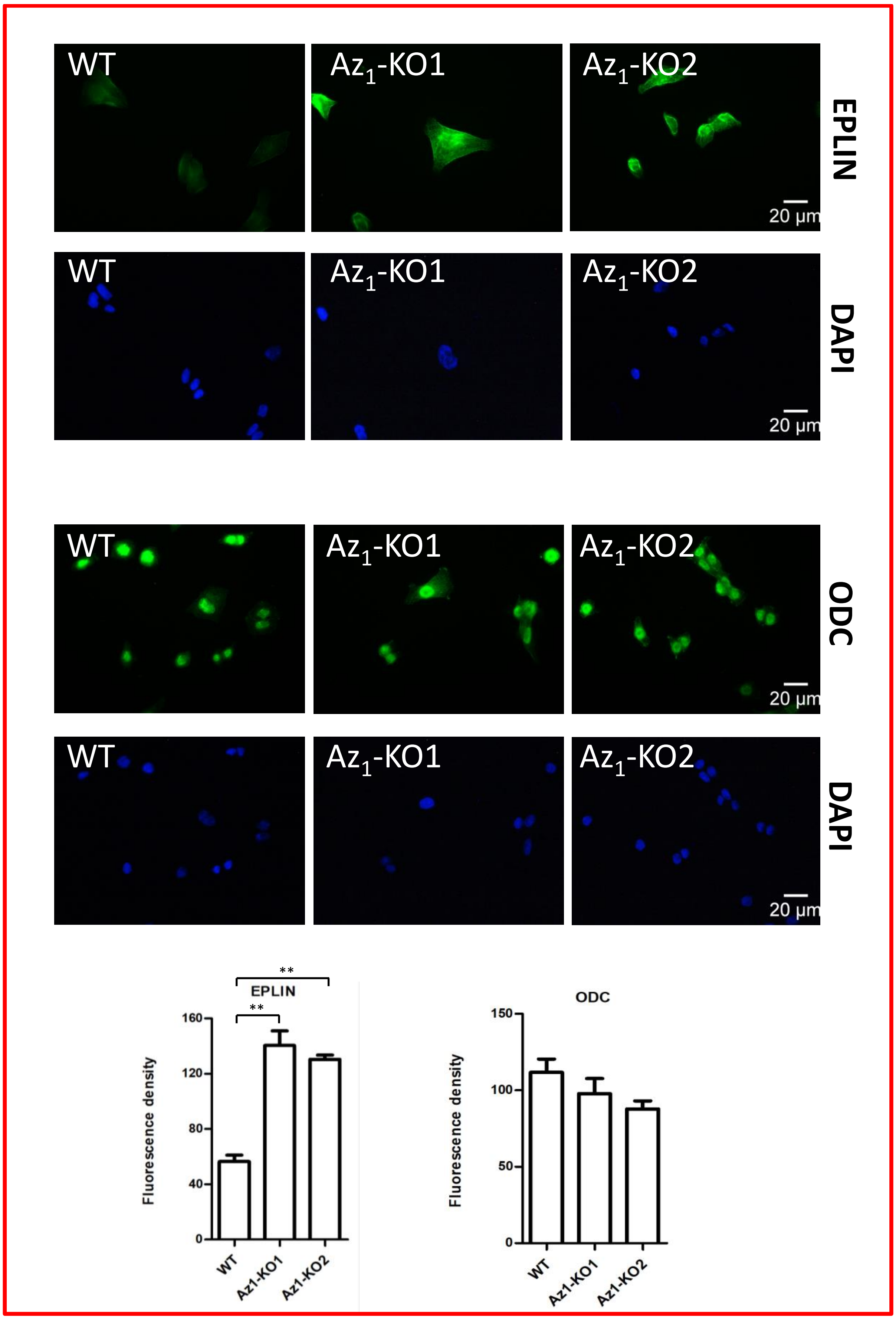

Fig. S13. Repeated images for Fig. S2

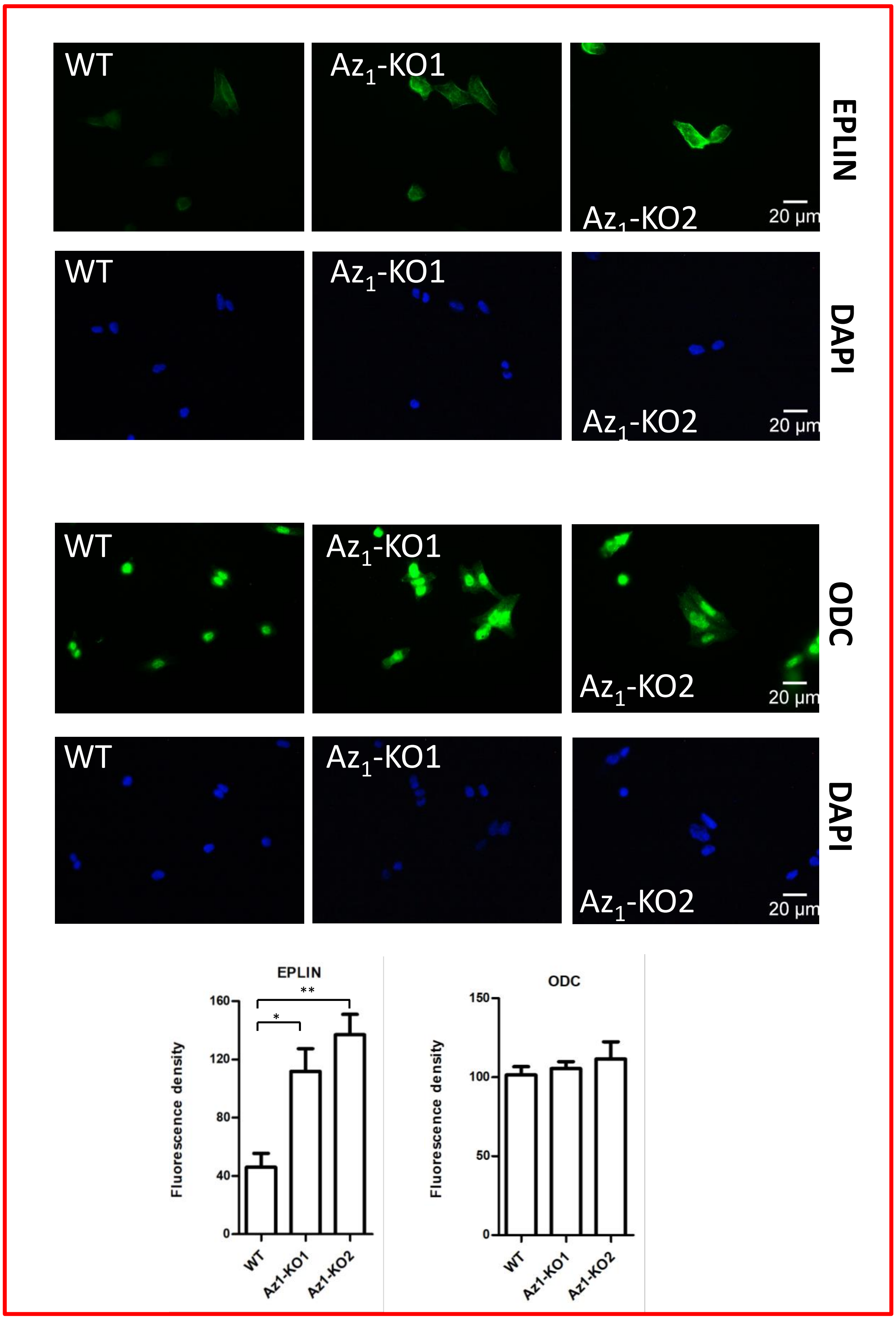

Fig. S13. Repeated images for Fig. S2
Repeated images for Fig. S2 are presented here along with the quantification results.

Table S1. Proteins identified by Mass Spectrometry

Full analysis for the KO/WT protein ratios.

	Candidates	H/L (WT/KO)	H/L (KO/WT)
1	Ornithine decarboxylase (ODC)	0.064829	7.6262
2	Apoptosis regulator BAX (Bax)	0.24292	3.962
3	Histone H2A type 2-C;Histone H2A type 2-A (HIST2H2AC;HIST2H2AA3)	0.68793	3.8151
4	Anaphase-promoting complex subunit 10 (ANAPC10)	0.64877	3.5153
5	Histone H2A.V;Histone H2A.Z;Histone H2A (H2AFV;H2AFZ))	0.52613	3.1963
6	LIM domain and actin-binding protein 1 (LIMA1)	0.33622	3.0333
7	Chromobox protein homolog 5 (CBX5)	0.37818	2.7435
8	Toll-interacting protein (TOLLIP)	0.2511	2.5699
9	DnaJ homolog subfamily A member 3, mitochondrial (DNAJA3)	0.66235	2.3044
10	Core histone macro-H2A.1;Histone H2A (H2AFY)	0.60171	2.2356
11	5-nucleotidase (NT5E)	0.26012	2.2349
12	Flotillin-2 (FLOT2)	0.6298	2.2063
13	Flotillin-1	0.65779	2.1251
14	Ras-related protein Rap-1b;Ras-related protein Rap-1b-like protein (RAP1B)	0.50543	2.1168
15	TRAF family member-associated NF-kappa-B activator (TANK)	0.40271	2.0871
16	SHC-transforming protein 1 (SHC1)	0.38465	1.9886
17	2-methoxy-6-polyprenyl-1,4-benzoquinol methylase, mitochondrial (COQ5)	0.50791	1.9142
18	Ras-related protein Rab-31 (RAB31)	0.6095	1.9012
19	Hypermethylated in cancer 2 protein (HIC2)	0.19705	1.8406
20	Guanine nucleotide-binding protein subunit beta-like protein 1 (GNB1L)	0.58536	1.8259
21	Amphiregulin (AREG)	0.11372	1.7692
22	Tumor necrosis factor ligand superfamily member 9 (TNFSF9)	0.25857	1.7227
23	Cytoplasmic phosphatidylinositol transfer protein 1 (PITPNC1)	0.43783	1.7158
24	Guanine nucleotide-binding protein G(i) subunit alpha-1 (GNAI1)	0.55379	1.6948
25	G1/S-specific cyclin-D1 (CCND1)	0.5011	1.6888
26	Epiplakin (EPPK1)	0.62731	1.6738
27	Protein XRP2 (RP2)	0.65856	1.6605
28	Targeting protein for Xklp2 (TPX2)	0.62788	1.6544
29	Solute carrier family 2, facilitated glucose transporter member 1 (SLC2A1)	0.31101	1.6226
30	Tripartite motif-containing protein 16;Tripartite motif-containing protein 16-like protein (TRIM16)	0.57017	1.6142
31	Caveolin-1;Caveolin (CAV1)	0.29744	1.6014
32	tRNA-dihydrouridine(47) synthase [NAD(P)(+)]-like (DUS3L)	0.53061	1.5866
33	Trophoblast glycoprotein (TPBG)	0.16225	1.5802

Table S2. Primer list

Primers for cloning the indicated plasmids are listed.

Name	Primer sequences from 5' to 3' (Forward)	Primer sequences from 5' to 3' (Reverse)
FLAG-EPLIN-β	aaa GGA TCC atg GACTACAAGGACGACGATGACAAG gaa tca tct cca ttt aat aga cgg	gc CTC GAG tcactcttcatcctcatcctcatcata
FLAG-EPLIN-α	aaa GGA TCC_atg GACTACAAGGACGACGATGACAAG gaaaattgtctaggagaatccagg	gc CTC GAG tcactcttcatcctcatcctcatcata
Delete_LIM domain	aaa GGA TCC atg GACTACAAGGACGACGATGACAAG gaa tca tct cca ttt aat aga cgg	gc CTC GAG tcttgcaggtgcctgaaacttctt
	aaa CTC GAG aagggcaactatgatgaaggcttt	gc CTC GAG tcactcttcatcctcatcctcatcata
HA-AZIN	aaa GGA TCC atg TAC CCA TAC GAT GTT CCA GAT TAC GCT aaa gga ttt att gat gat gca aac	gc CTC GAG tta agc ttc agc gga aaa gct gtc
FLAG-ODC	aaa GGA TCC atg GAC TAC AAG GAC GAC GAT GAC AAG aac aac ttt ggt aat gaa gag	aaa CTC GAG cta cac att aat act agc cga
EPLIN-shRNA	CCGG—TTACTTCTCTTCTTCTCCATG—CTCGAG—CATGGAGAAGAAGAGAAGTAA—TTTTTG	AATTCAAAAA—TTACTTCTCTTCTTCTCCATG—CTCGAG—CATGGAGAAGAAGAGAAGTAA

Table S3. Antibody dilutions

The dilution factors of all the antibodies used in this paper are listed.

Antibodies	Dilutions
Actin	1: 10000
ODC	1: 500
Flag	1: 2000
EPLIN	1: 400
Myc	1: 1000
HA-tag	1: 1000
His-tag	1: 200
Mouse secondary antibody	1: 3000
Rabbit secondary antibody	1: 3000

Journal of Cell Science • Supplementary information
